# An *in vivo *RNA interference screen identifies gene networks controlling *Drosophila melanogaster *blood cell homeostasis

**DOI:** 10.1186/1471-213X-10-65

**Published:** 2010-06-11

**Authors:** Amélie Avet-Rochex, Karène Boyer, Cédric Polesello, Vanessa Gobert, Dani Osman, Fernando Roch, Benoit Augé, Jennifer Zanet, Marc Haenlin, Lucas Waltzer

**Affiliations:** 1 Université de Toulouse, UPS, CBD (Centre de Biologie du Développement), Bât4R3, 118 route de Narbonne, 31062 Toulouse, France; 2 CNRS, CBD UMR5547, 31062 Toulouse, France; 3 King's College London, Guy's Campus, London SE1 1UL, UK

## Abstract

**Background:**

In metazoans, the hematopoietic system plays a key role both in normal development and in defense of the organism. In Drosophila, the cellular immune response involves three types of blood cells: plasmatocytes, crystal cells and lamellocytes. This last cell type is barely present in healthy larvae, but its production is strongly induced upon wasp parasitization or in mutant contexts affecting larval blood cell homeostasis. Notably, several zygotic mutations leading to melanotic mass (or "tumor") formation in larvae have been associated to the deregulated differentiation of lamellocytes. To gain further insights into the gene regulatory network and the mechanisms controlling larval blood cell homeostasis, we conducted a tissue-specific loss of function screen using hemocyte-specific Gal4 drivers and *UAS-dsRNA *transgenic lines.

**Results:**

By targeting around 10% of the Drosophila genes, this *in vivo *RNA interference screen allowed us to recover 59 melanotic tumor suppressor genes. In line with previous studies, we show that melanotic tumor formation is associated with the precocious differentiation of stem-cell like blood progenitors in the larval hematopoietic organ (the lymph gland) and the spurious differentiation of lamellocytes. We also find that melanotic tumor formation can be elicited by defects either in the fat body, the embryo-derived hemocytes or the lymph gland. In addition, we provide a definitive confirmation that lymph gland is not the only source of lamellocytes as embryo-derived plasmatocytes can differentiate into lamellocytes either upon wasp infection or upon loss of function of the Friend of GATA cofactor U-shaped.

**Conclusions:**

In this study, we identify 55 genes whose function had not been linked to blood cell development or function before in Drosophila. Moreover our analyses reveal an unanticipated plasticity of embryo-derived plasmatocytes, thereby shedding new light on blood cell lineage relationship, and pinpoint the Friend of GATA transcription cofactor U-shaped as a key regulator of the plasmatocyte to lamellocyte transformation.

## Background

In metazoan, blood cells play a critical role in establishing the proper response against invading pathogens or in removing both cancerous and apoptotic cells [1]. Conversely, deregulations of the hematopoietic differentiation program are at the origin of numerous pathologies, including leukemia and auto-immune diseases [2]. As many key signaling pathways and transcription factors controlling blood cell development and functions have been conserved from humans to Drosophila [3], this organism has emerged as an attractive model to investigate the genetic basis controlling blood cell homeostasis.

Drosophila hematopoiesis occurs in two spatially and temporally distinct phases. In the early embryo, blood cell progenitors (prohemocytes) arise from the head mesoderm [4]. These hemocytes subsist in the larva either in circulation in the hemolymph or attached to the inner surface of the integument, forming the so-called sessile islands that can be mobilized upon infection [5,6]. A second hematopoietic wave occurs in the larva in a specialized organ called the lymph gland [5]. In third instar larvae, the lymph gland is composed of a pair of primary lobes and several more posterior secondary lobes. Each primary lobe is subdivided into three zones: (1) the cortical zone, containing differentiated hemocytes; (2) the medullary zone, containing prohemocytes; and (3) the posterior signaling center, a small group of cells whose activity is required to maintain medullary zone cells into a progenitor state [7-9]. The smaller posterior lobes, presenting no organized structure, consist mainly of prohemocytes [7]. In normal conditions, both the circulating and sessile cells in the larva are only of embryonic origin [6] whereas hemocytes from the lymph gland are released into circulation only at pupariation [5]. Finally, in the adult, no hematopoietic tissue has been described and hemocytes of both embryonic and lymph gland origin are observed [6].

Prohemocytes give rise to three terminally differentiated cell types: plasmatocytes, crystal cells and lamellocytes [3]. Plasmatocytes, which comprise 90-95% of the larval circulating blood cells, are phagocytic cells that engulf apoptotic bodies and pathogens [10-13]. Crystal cells secrete components of the melanization cascade, an insect-specific immune reaction involved in wound healing and in the encapsulation of large foreign bodies [14-16]. Lamellocytes are large flattened non-phagocytic cells normally scarcely present in the larva but their development is massively induced upon certain immune challenges such as infection of the larvae by eggs of the parasitoid wasp *Leptopilina boulardi *[17]. Parasitization elicits lymph gland overgrowth, massive production of lamellocytes, and precocious hemocyte release from the lymph gland into the circulation. Together with the other blood cell types, lamellocytes form a melanotic capsule around the parasitoid egg to prevent its development [5,18]. While it was initially proposed that lamellocytes might represent an ultimate state of plasmatocyte differentiation [17,19-21] further evidence suggests that they derive only from lymph gland progenitors [5,8,18,22,23]. Yet this view has been recently challenged as cells from the sessile islands were shown to differentiate into lamellocytes after wasp infection [24].

Interfering with normal blood cell development and/or function can trigger an aberrant immune response characterized by lymph gland overgrowth and massive differentiation of lamellocytes [25]. This response culminates with the premature disintegration of the lymph gland and the formation in the larvae of "melanotic tumors" (also called melanotic masses, melanotic nodules or pseudotumors) constituted by melanized aggregates of hemocytes, mostly lamellocytes, sometimes surrounding cells from other tissues. Melanotic masses are easily observable through the larval cuticle and a large number of « melanotic tumor suppressor genes », were identified based on such phenotype [25]. Unfortunately, the nature of the mutated gene has not been ascertained in the majority of the cases and the contribution of blood cells to the phenotype has seldom been evaluated [26]. Notwithstanding, available evidences suggest that presence of melanotic tumors reflects defects in the hematopoietic developmental program and/or in the immune surveillance of self-tissues. Accordingly, melanotic mutations have been classically subdivided in two categories [25,27]: (1) class I mutations modify a non-hematopoietic tissue and induce a kind of "autoimmune response", as the mutations in *kurtz *or *spaghetti *[26,28], (2) class II mutations affect internal regulatory pathways within the hemocytes themselves, such as gain of function mutations in JAK/STAT or Toll signaling pathway [29-34]. Hence, melanotic tumor suppressor genes are potential candidates for regulating both hematopoiesis and blood cell function.

In this work, we conducted a large-scale screen for melanotic tumor suppressor genes aimed specifically at the identification of genes involved in blood cell homeostasis, taking advantage of recently developed *UAS-dsRNA *transgenic line collections. Down-regulation of the targeted genes was specifically induced in the blood cells or both in the blood cells and the fat body using different Gal4 drivers. By individually inactivating the function of around 10% of the Drosophila genes, we recovered 59 melanotic tumor suppressor genes. This approach allowed us to pinpoint several new pathways controlling blood cell homeostasis. By analyzing some of these candidates, we further demonstrate that melanotic masses can be induced by defects in a specific subset of cells and demonstrate that embryonic-derived plasmatocytes can differentiate into lamellocytes.

## Results

### A loss of function screen for melanotic tumor suppressor genes

To identify new genes regulating Drosophila blood cell homeostasis, we performed a screen for melanotic tumor suppressor genes (*i.e*. genes whose loss of function induces melanotic mass formation in third instar larvae). For this, we used of a collection of RNAi transgenes (*UAS-dsRNA*) that consist of short gene fragments (300-500 bp) cloned as inverted repeats and expressed via the binary Gal4/UAS system http://www.shigen.nig.ac.fly/nigfly, thus allowing tissue-specific gene knock-down. We took advantage of this collection to induce RNAi in blood cells using three different drivers: *srp-Gal4, cg-Gal4*, and *hml*Δ-*Gal4 *[35-37]. *spr-Gal4 *is expressed in all the embryo-derived hemocytes from the early embryonic stages, as well as in the larval lymph gland and fat body [35]. *cg-Gal4 *is expressed in the plasmatocytes both in the late embryo and in the larvae, including in the lymph gland cortical zone, and also drives at high levels in the larval fat body [7,36]. *hml*Δ-*Gal4 *is expressed only at the larval stages, in almost all the circulating blood cells and in the cortical zone of the lymph gland [38]. All together these three drivers, whose expression patterns are illustrated in Additional file 1, Figure S1, allow targeting most of the tissues involved in hematopoietic development and cellular immunity in Drosophila.

**Figure 1:**
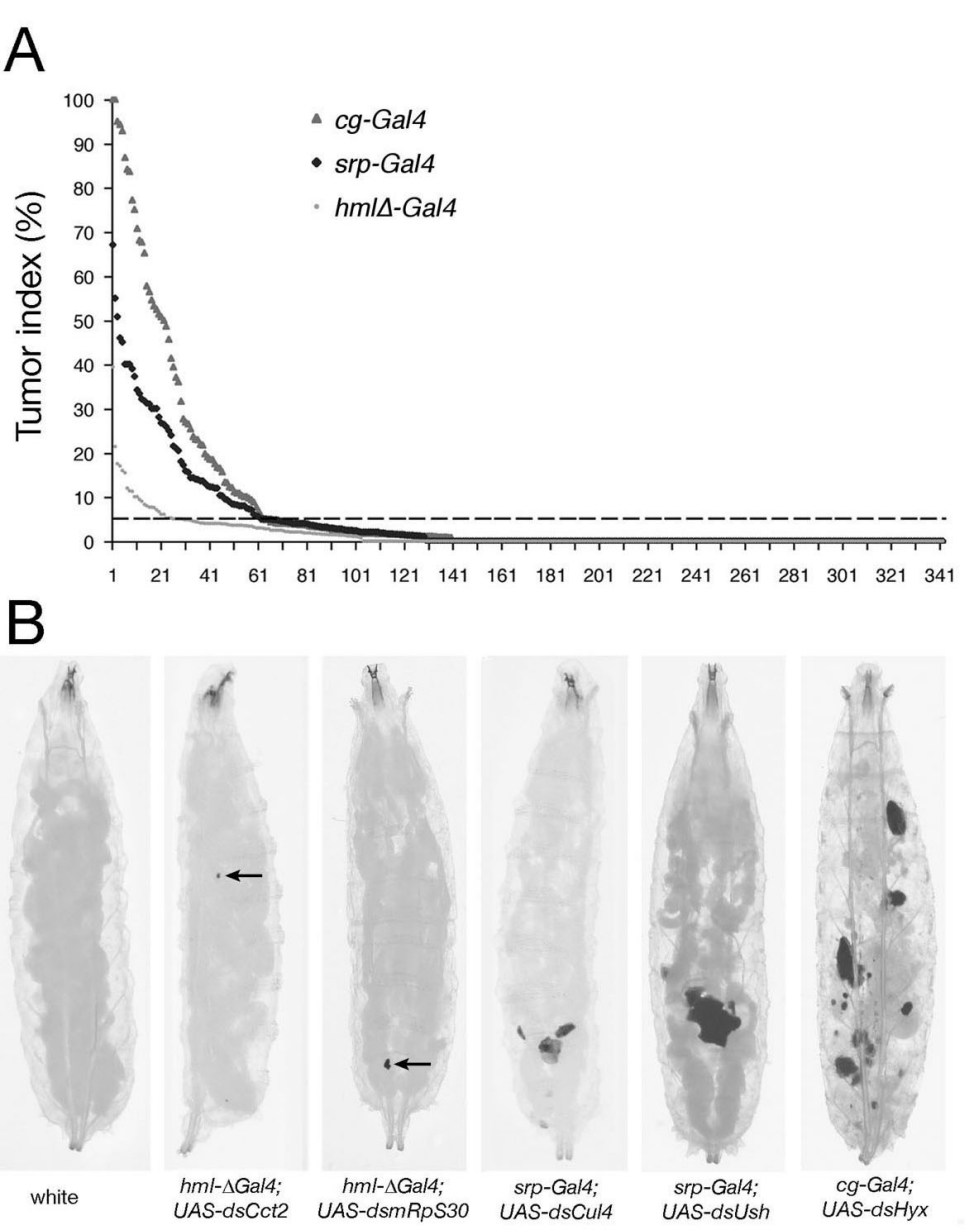
**An RNAi screen for melanotic suppressor genes**. (A) Distribution of tumor indices for the 342 UAS-dsRNA lines retested with the three drivers (*hml*Δ-*Gal4, srp-Gal4, cg-Gal4*). For each of the three driver lines, the candidates are classified by decreasing tumor index (% of larvae carrying at least one melanotic nodule). The dotted line indicates the 5% threshold that we used to select 96 candidates for secondary validations. (B) Example of melanotic mass mutant phenotypes recovered in the screen. Third instar larvae are shown with their corresponding genotypes. Small melanotic masses are indicated by an arrow. Note the dissociation of the fat body in the *cg-Gal4; UAS-ds-hyx *larva.

Based on pilot experiments, our screen was first performed with *srp-Gal4 *and *hml*Δ-*Gal4 *on *UAS-dsRNA *transgenes predicted to target 1341 of the 13825 predicted protein-coding genes of Drosophila (Drosophila genome release 5; http://flybase.org) (Additional file 4, Table S1). All the hits were subsequently retested with the three drivers on a higher number of larvae. Given that all together 2.5% of the ± 145000 larvae that we screened had melanotic nodules, we selected as positives only those lines that scored two folds above this baseline, *i.e*. with a tumor index ≥5% (Figure 1A). Thereby we identified 96 genes whose RNAi-induced down-regulation with one of the three drivers induced melanotic masses in at least 5% of the emerging larvae (Additional file 5, Table S2). Interestingly four of these genes (*cactus*, *DREF*, *ush *and *ND75*) were already known to be implicated in melanotic mass formation and/or lamellocyte differentiation [39-42], thereby validating our screening strategy. Figure 1B displays some representative larvae harboring melanotic tumors that we obtained in the screen. We did not consider melanotic spots (on the cuticle, gut, trachea....) as genuine melanotic tumors since they were shown to arise independently of a modification in larval blood cell homeostasis [26]. While smaller nodules were circulating freely in the hemocel, larger ones were most often localized to the posterior part of the larvae. In some rare cases, we observed lymph gland melanization or disintegration of the fat body (Figure 1B, right most panel). Moreover, for a given gene, the penetrance of the phenotype is largely dependent on the Gal4 line (Additional file 5, Table S2). Indeed, the median tumor index for the 96 candidates was 1.5% with *hml*Δ-*Gal4*, 9% with *srp-Gal4 *and 19% with *cg-Gal4*. Of note, *cg-Gal4 *also caused severe growth delay or lethality before the third instar larval stage with a few candidates, which impaired the analysis of melanotic mass formation in such cases and excluded its use in the screen first step (Additional file 5, Table S2).

### Validation of the candidates

**Figure 2:**
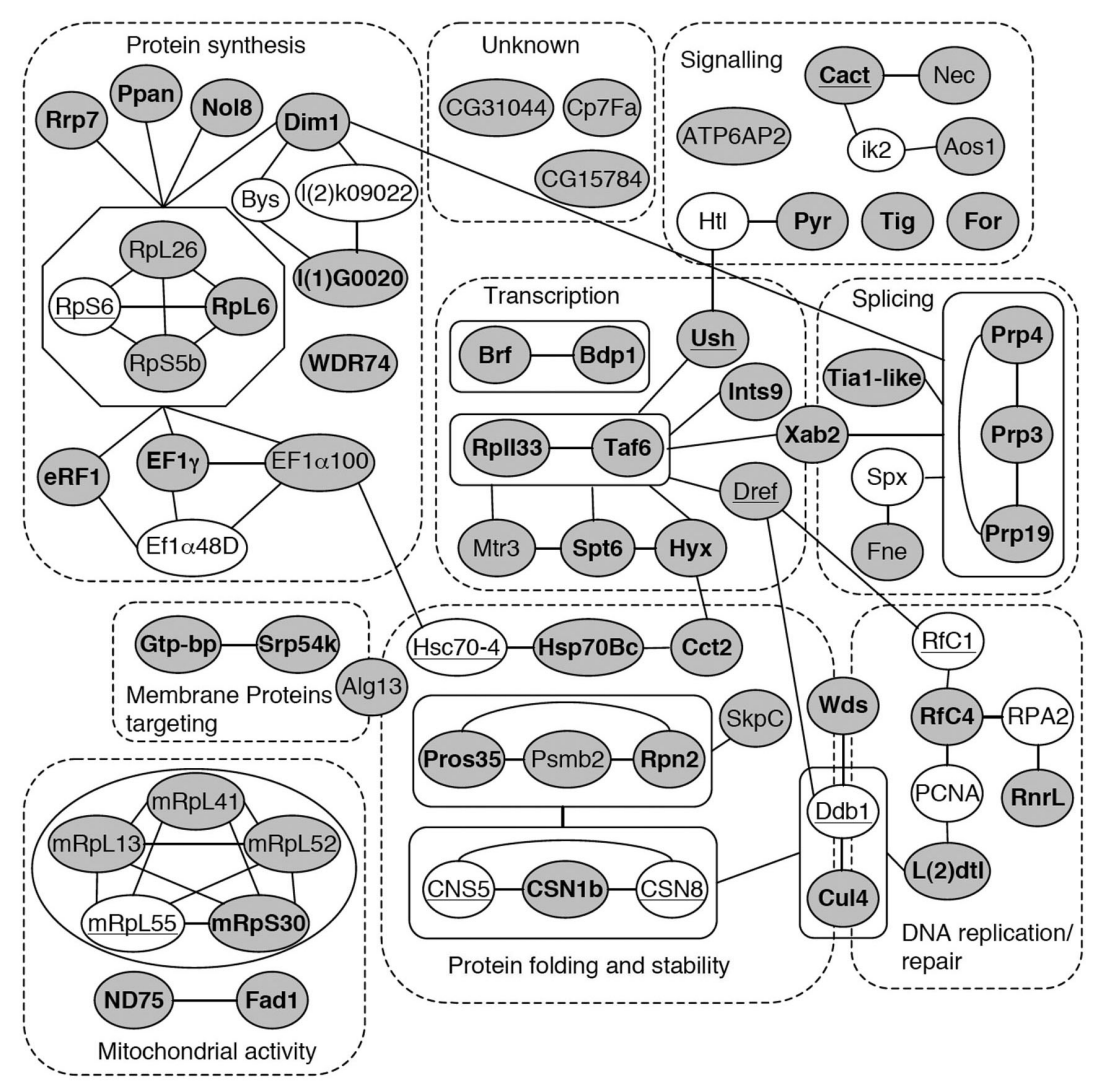
**An interaction network of the melanotic suppressors**. The 59 confirmed melanotic suppressors identified in the screen are depicted as grey nodes together with their (inferred) name. Additional factors not identified in the screen but linking two or more melanotic suppressors are represented as white nodes. Factors previously associated with melanotic mass development and/or lamellocyte differentiation are underlined. Candidates that were confirmed using non-overlapping dsRNA are indicated in bold. The different melanotic suppressors are grouped in 9 categories (dashed lines) based on GO annotation and data mining. Factors belonging to a well-defined molecular complex are boxed together. Physical or functional interactions between the different factors are represented by edges.

Expression of long double stranded RNA can cause non-specific phenotypes due to off-target effects (OTE) [43]. A common measure of dsRNA targeting specificity is the specificity score, S19, which is the number of all on-target 19-mer matches divided by the total number of matches of a given RNAi hairpin [43]. Among the candidates we isolated, 43 of the 96 hairpins tested had no predicted off-target (S19 = 1), 25 had a S19 above 0.99, 22 between 0.99 and 0.8 and 16 below 0.8, suggesting that the vast majority of the dsRNA we used were specific (Additional file 5, Table S2). Besides OTE, another potential source of false positives is the dsRNA transgene insertion itself, which might interfere with expression of nearby genes and produce melanotic nodules. To validate our hits, we obtained independent secondary *UAS-dsRNA *lines for the entire set of candidate genes, except two for which no secondary lines were available: *cactus*, a negative regulator of the Toll pathway well known as a melanotic tumor suppressor gene [32,34,40], and CG9663, which codes for an ABCG transporter. Of note, 59 of these 94 secondary UAS-dsRNA targeted a non-overlapping sequence in the candidate gene mRNA as compared to the original set (Additional file 5, Table S2). Using these secondary RNAi lines, we could phenocopy formation of melanotic masses for 58 genes, including 40 of them (70%) using non-overlapping dsRNA (Additional file 6, Table S3). Among the 18 genes where overlapping dsRNA were used, mutant alleles for two of them induced melanotic tumor formation (*Dref and Aos1*) and 10 code for proteins that interact with an other melanotic suppressors validated by non-overlapping dsRNA or genetic means (see below and Figure 2). As for the 6 remaining genes in that category (CG14512, CG15784, CG31044, CG8444, *Cp7Fa *and *fne*), we cannot exclude an off-target effect although dsRNA lines targeting CG14512, CG8444 and *fne *have an S19 score of 1 and above 0.9 for CG15784 and *Cp7Fa*. Hence, together with *cactus*, our screen allowed us to identify 59 genes potentially controlling larval blood cell homeostasis.

We also sought to validate these candidates by checking the phenotypes of genetic mutants affecting their activity. However, in most cases, no mutants have been described for these genes or the available mutations result in lethality before the third instar larval stage, thereby precluding this kind of analysis. Nonetheless, beside the already described mutations in *cactus *[32,34,40], we could confirm presence of melanotic nodules in larvae carrying zygotic mutations for five genes: *u-shaped *(previously associated only to lamellocyte differentiation) [41], *pyramus*, *RfC4*, *tiggrin *and *Aos1 *(Additional file 6, Table S3). These results support the idea that the use of *UAS-dsRNA *allows efficient identification of a large panel of genes participating in blood cell development.

### Identification of gene networks controlling blood cell homeostasis

To gain insights into the putative functions of the candidate genes and into the different pathways regulating blood cell homeostasis, we built an interaction network between the candidates. Accordingly, we searched for high confidence yeast two-hybrid, biochemical or genetic interactions data using various databases (DroID, BioGrid, Flybase....) as well as manual text mining for each of the 59 genes and their mammalian or yeast orthologs. We only considered first order (direct) and second order (through one intermediate) interactions between genes contained in our hit list or previously identified as melanotic tumor suppressors. This approach allowed us to uncover several nodes of interactions between them (Figure 2) as 47 of the candidates are linked to at least one other gene in the network. Interestingly, 14 of the candidate genes code for proteins that are part of complexes with previously described melanotic tumor suppressors, thereby confirming that they are genuine melanotic tumor suppressor genes. Namely, these 14 genes code for the cytoplasmic ribosomal constituents RpL26, RpL6 and RpS5b, which associate with RpS6 [44], the mitochondrial ribosomal constituents mRpL13, mRpL41, mRpL52 and mRpS30, which bind to mRpL55 [45], the COP9 signalosome component CSN1b, which associates with CSN5 and CSN8 [46,47], Hsp70Bc and EF1α (100)E, which are linked to Hsc70-4 [48], the replication factor RfC4, which associates with RfC1 [49], as well as Wds, l(2)dtl and Cul4, which bind to DDB1 [50]. In addition, it is worth noting that DREF was shown to regulate the transcription of two melanotic suppressors, *ddb1 *and *rfc1*, in cell culture [49,50]. Finally, several candidates are indirectly connected to such melanotic suppressors. For instance, the translation factors (EF1α(110), EF1γ and eRF1) or the factors involved in ribosome assembly (Dim1, Nol8, l(1)G0020, Rrp7 and PPAN) are functionally linked to RpS6 function.

Given that our screen allowed identification of several sets of interconnected genes, we asked whether conversely known partners of the genes we identified also behave as melanotic tumor suppressor genes. As a root, we chose Cct2, a component of the well defined and evolutionarily conserved chaperonin complex TriC/CCT (TCP1-ring Complex or Chaperonin Containing TCP1) [51], which had not been linked to melanotic mass development before. The CCT complex is composed of eight subunits (Tcp1 and Cct2-8) and is a cytosolic chaperonin complex regulating protein folding. We obtained UAS-dsRNA lines against 5 other CCT subunits (Tcp1, Cct4, Cct5, Cct7 and Cct8) and tested their capacity to induce melanotic nodules upon expression under the control of the three hematopoietic drivers used in the screen. As summarized Table 1, downregulation of any of the six CCT components tested induced melanotic masses. These results show that the CCT complex plays a pivotal role in regulating larval blood cell homeostasis and indicate that the different candidates from the screen can be used as entry points to explore the network of genes implicated in melanotic mass formation.

**Table 1:** Melanotic masses induction upon loss of CCT complex component

			*UAS-dsRNA *driver		
			
CG	SYMBOL	S19	*srp-Gal4*	*hmlΔ-Gal4*	*cg-Gal4*
CG7033	Cct2	1	+++	-	++ *^a^*
CG5525	Cct4	0.99	+++	-	++++ *^a^*
CG8439	Cct5	0.99	+++	+	++ *^a^*
CG8351	Cct7	0.99	++++	-	+++
CG8258	Cct8	0.99	++	-	+++
CG5374	TCP1	0.99	+++	++	+++ *^a^*

Melanotic masses indices: (-) < 5%; (+) 5-10%; (++) 10-20%; (+++) 20-50%; (++++) > 50%.

*^a ^*Dead second and third instar larvae were observed and no or only few adults emerged.

For further analysis of melanotic tumor formation process, we focused our attention on 5 genes that induced melanotic mass to high frequency and might represent different classes of melanotic tumor suppressor genes: *cct2*, *cul4*, *hyx*, *mRpS30 *and *ush*.

### Melanotic masses are associated to lamellocyte production and premature lymph gland differentiation

**Figure 3:**
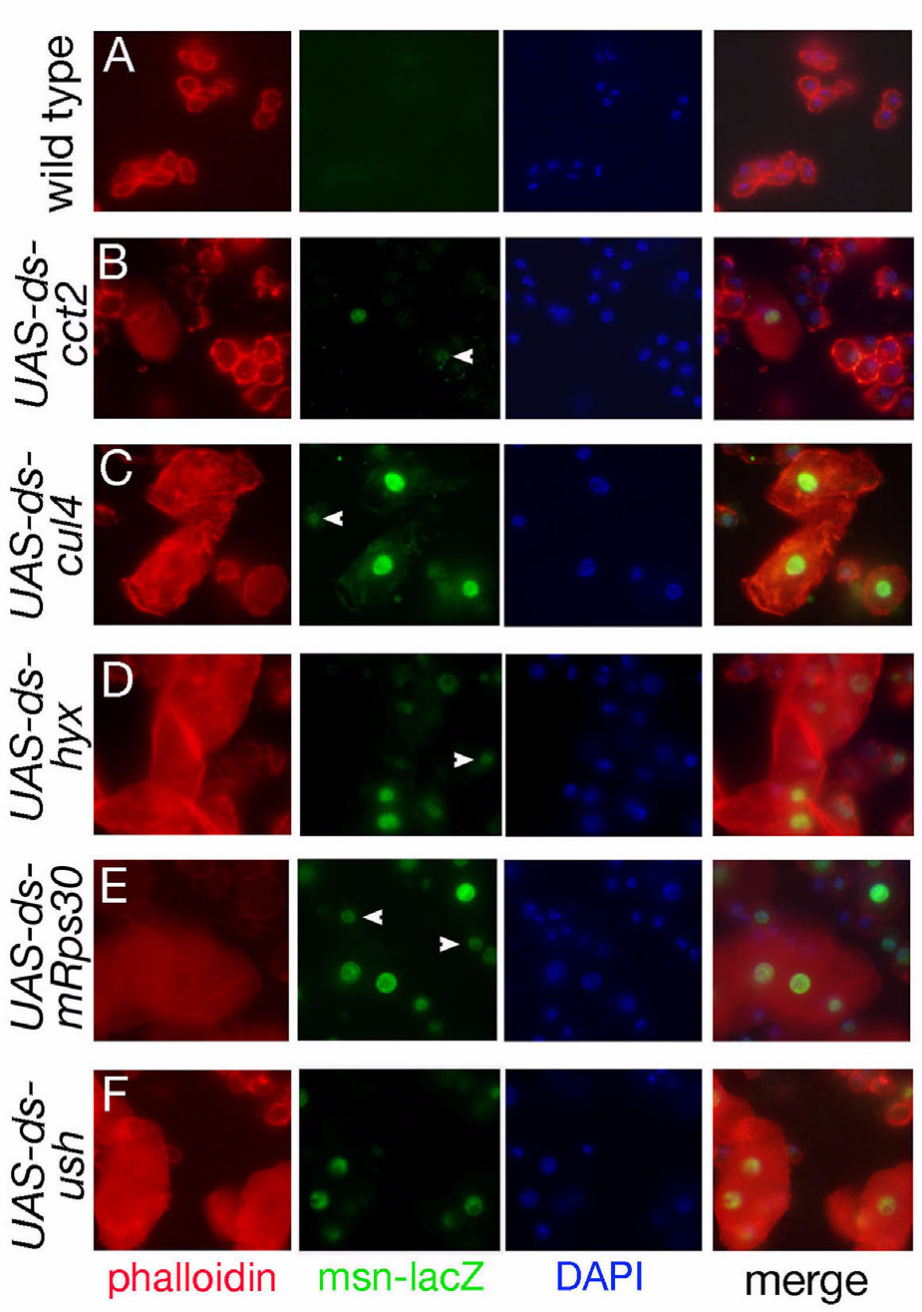
**Blood cell phenotypes associated to melanotic mass formation**. Blood smears from third instar larvae carrying the *msn-lacZ *transgene and expressing *UAS-dsRNA *targeting the indicated gene under the control of *srp-Gal4*. Hemocyte actin cytoskeleton was visualized using phalloidin (red) and expression of the lamellocyte marker *msn-lacZ *was revealed by fluorescent immunolabeling against β-Gal (green). Nuclei were stained with DAPI. Arrowheads indicate atypical hemocytes that express β-Gal but do not exhibit the large flattened morphology of lamellocytes.

To confirm that the melanotic masses we observed arise from a modification in larval blood cell homeostasis, we bled larvae expressing dsRNA for *cct2, cul4, hyx, mRpS30 *and *ush *and looked for lamellocyte differentiation. Figure 3 shows representative bleeds obtained from third instar larvae expressing the indicated dsRNA under the control of *srp-Gal4 *(similar results were obtained with *hml*Δ-*Gal4 *and *cg-Gal4*, data not shown). Lamellocytes are normally absent in healthy larvae and can be easily distinguished from other blood cell types based on their morphology (large flattened cells) and the expression of high levels of actin as well as specific markers such as *msn-lacZ *and α-*ps4 *[5,52,53]. Phalloidin staining and morphological examination of larval blood smears showed that all the larvae with melanotic nodules contained numerous lamellocytes in circulation, whereas this blood cell type was scarcely found in wild type controls. As expected, *msn-lacZ *was strongly expressed in the induced population of lamellocytes. In addition, we observed a reproducible, albeit weaker, expression of *msn-lacZ *in smaller blood cells unveiling a β-gal^+ ^population that is not present in wild type larvae. These results suggest that melanotic tumor formation is also associated to the activation of *msn-lacZ *in plasmatocytes or in circulating lamellocyte progenitors (see below). Finally, analysis of the melanotic masses themselves confirmed that they contained numerous lamellocytes (Additional file 2, Figure S2).

**Figure 4:**
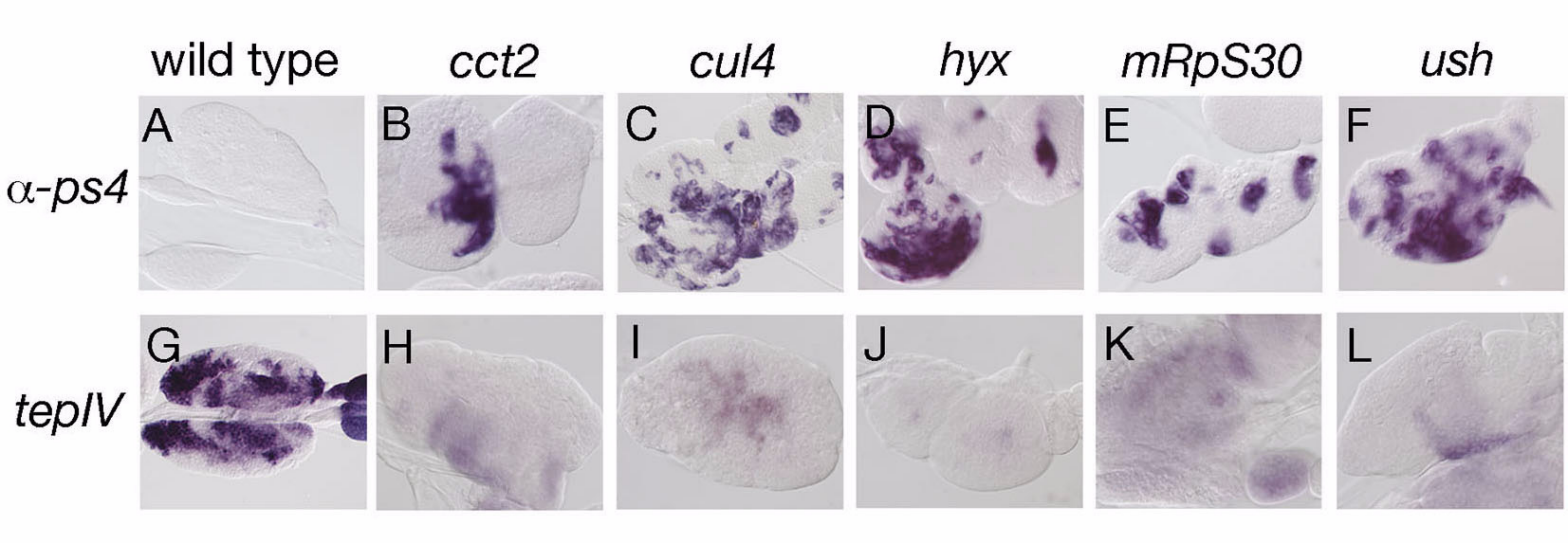
**Lymph gland differentiation status**. Expression of the lamellocyte differentiation marker α-*ps4 *(A-F) or of the prohemocyte marker *tepIV *(G-L) was revealed by *in situ *hybridization on lymph glands from early third instar larvae expressing *UAS-dsRNA *targeting the indicated gene under the control of *srp-Gal4*.

We also monitored lymph gland differentiation status. Consistent with previous reports, we observed precocious disintegration of the lymph gland in most larvae harboring melanotic masses. By collecting larvae without macroscopically apparent or with smaller nodules, we were able to recover intact lymph gland and found that, contrary to wild type, larvae expressing *UAS-dsRNA *contained lamellocytes in their lymph glands, as revealed by *in situ *hybridization against α-*ps4 *(Figure 4A-F). In addition, *tepIV *staining revealed that the medullary zone, which contains stem-like blood progenitors, was markedly reduced or absent (Figure 4G-L). Thus, similar to wasp-egg parasitization [8,18,22], dsRNA-induced capsule formation affect larval blood cell homeostasis by activating lamellocyte development and premature differentiation of lymph gland prohemocytes.

### Melanotic capsule formation is induced in response to defect in specific tissues

The three Gal4 drivers we used in our screen are predominantly expressed in blood cells, but they are also expressed in other territories (*e.g*. the fat body for *srp-Gal4 *and *cg-Gal4*) and in overlapping patterns within the hematopoietic lineages (embryonic and larval hemocytes for *srp-Gal4*, differentiated embryonic and larval plasmatocytes for *cg-Gal4*, differentiated larval hemocytes for *hml*Δ-*Gal4*). We thus asked whether melanotic nodule formation reflected a general response to a defect in any tissue or was directly elicited by the modification of a (particular) blood cell type. To investigate this issue, we used a battery of Gal4 lines that are either not expressed in hematopoietic tissues (*fb-Gal4, cad-Gal4, repo-Gal4, elav-Gal4, MS1096*) or in restricted hematopoietic compartments (*sn-Gal4, gcm-Gal4, tepIV-Gal4*), or in overlapping patterns with the previous drivers (*sn-Gal4, gcm-Gal4, tepIV-Gal4, fb-Gal4, cad-Gal4*) (Table 2). These different drivers were used to assess through which cell type the targeted loss of *cct2, cul4, hyx, mRps30 *or *ush *can induce melanotic tumors.

**Table 2:** Melanotic masses and lamellocytes are induced in response to defects in specific tissues

			UAS-dsRNA				
			
driver	expression pattern	wild type	*cct2*	*cul4*	*Hyx*	*mRpS30*	*ush*
srp-Gal4	EH, LH, LG, FB	- / -	+ / +	+ / +	+ / +	+ / +	+ / +
cg-Gal4	EH, LH, LG (CZ), FB	- / -	+ / +	+ / +	+ / +	+ / +	+ / +
hmlΔ-Gal4	LH, LG (CZ), HG	- / -	+ / +	+ / +	+ / +	+ / +	+ / +
sn-Gal4	EH	- / -	- / -	+ / +	+ / +	- / -	+ / +
gcm-Gal4	EH, GC	- / -	+ / +	+ / +	+ / +	- / -	- / +
tepIV-Gal4	LG (MZ), CNS	- / -	+ / +	+ / +	Lethal	- / -	- / -
fb-Gal4	FB, WD, CNS	- / -	- / -	+ / +	- / -	+ / +	- / -
cad-Gal4	HG	- / -	- / -	- / -	- / -	- / -	- / -
repo-Gal4	GC	- / -	- / -	- / -	- / -	- / -	- / -
elav-Gal4	CNS, PNS	- / -	lethal	- / -	Lethal	lethal	- / -
MS1096	WD	- / -	- / -	- / -	- / -	- / -	- / -

CNS: central nervous system, HG: hindgut, PNS: peripheral nervous sytem, CZ: cortical zone, EH: embryonic hemocytes; FB: fat body, GC: glial cells, LH: larval hemocytes, LG: lymph gland, MZ: medullary zone, WD: wing disc. - / -: absence of melanotic masses or lamellocytes;+ /+: presence of melanotic masses and lamellocytes; - / +: rare melanotic masses but presence of lamellocytes.

**Figure 5:**
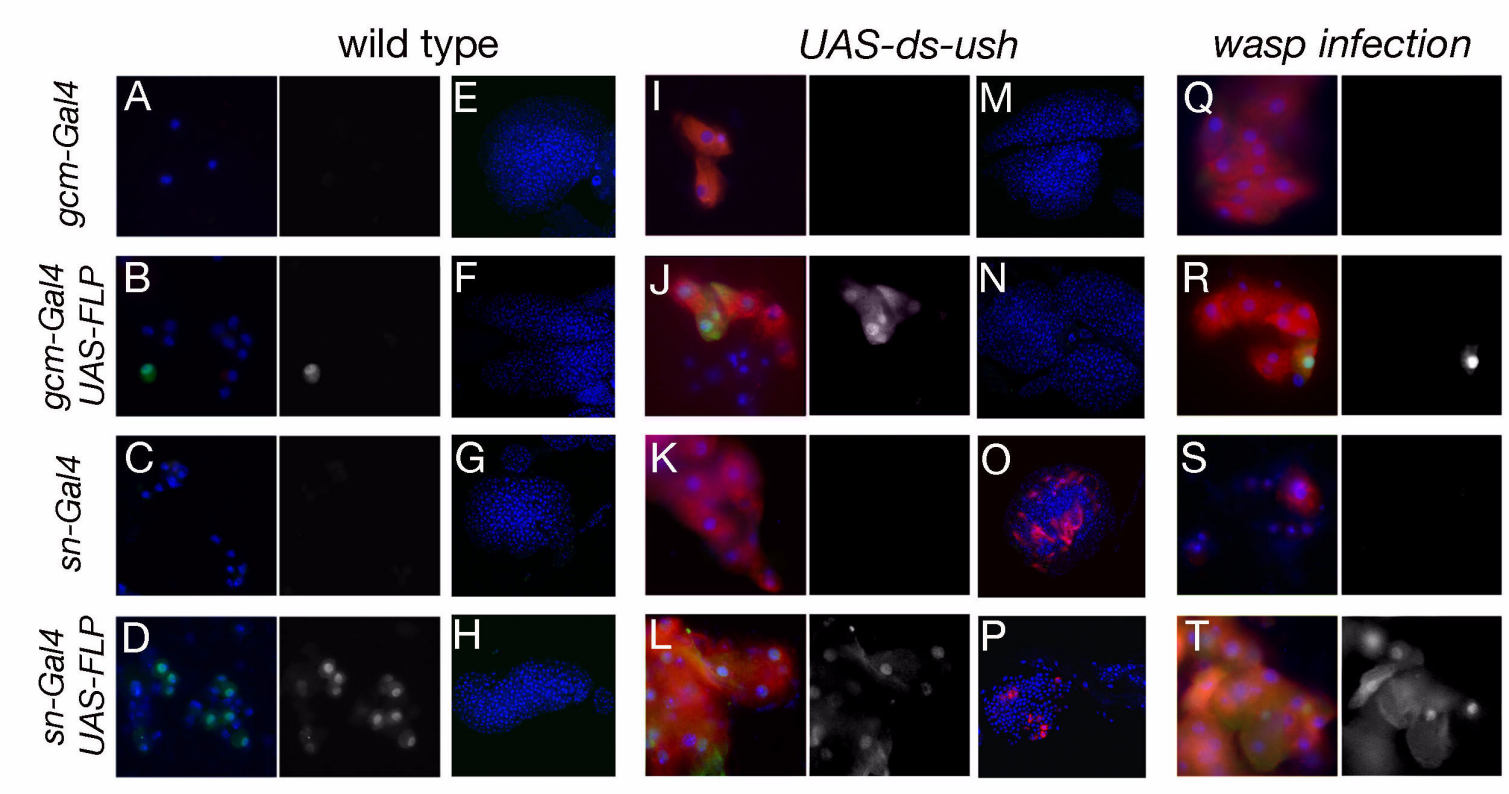
**Fate of embryonic blood cells**. (A-T) Blood smears (A-D, I-L and Q-T) and lymph glands (E-H and M-P) of third instar larvae. The *Act > FRT > CD2 > FRT > GAL4 *cassette and the *UAS-FLP *and *UAS-GFP *transgenes were used to permanently label the cells that express *sn-Gal4 *or *gcm-Gal4*. Immunostaining against GFP (green, also displayed in white on right panel of each blood smear) was used to monitor *gcm-Gal4, UAS-GFP *or *sn-Gal4, UAS-GFP *expression. *In situ *hybridization against α-*ps4 *(red) was used to reveal lamellocyte differentiation. Nuclei were counterstained with DAPI. GFP labeling alone is shown to the right. (A-H) larvae raised in wild-type conditions, (I-P) larvae carrying a *UAS-dsRNA *transgene against *ush*, (Q-T) larvae submitted to parasitization by *L. boulardi*. (A, E, I, M, Q) *gcm-Gal4, UAS-GFP; Act > FRT > CD2 > FRT > GAL4; *(B, F, J, N R) *UAS-FLP; gcm-Gal4, UAS-GFP; Act > FRT > CD2 > FRT > GAL4; *(C, G, K, O, S) *sn-Gal4, UAS-GFP; Act > FRT > CD2 > FRT > GAL4; (D, H, L, P, T) UAS-FLP; sn-Gal4, UAS-GFP; Act > FRT > CD2 > FRT > GAL4;*

We could induce both melanotic nodules and lamellocyte differentiation by targeting *cul4 *or *cct2 *dsRNA specifically in the lymph gland prohemocytes with the *tepIV-Gal4 *line (see Additional file 1, Figure S1 for its expression pattern), indicating that these genes may participate in blood cell progenitor fate maintenance and/or restrict their differentiation potential. Interestingly, targeting dsRNA expression only in plasmatocytes during embryogenesis either with *sn-Gal4 *[[54], Zanet *et al.*, in preparation] or *gcm-Gal4 *[35] (which are not maintained in larval hemocytes, see Figure 5 and Additional file 1, Figure S1) induced lamellocyte differentiation and melanotic mass formation in the case of *ush, hyx, cct2 *and *cul4*. Therefore, loss of function restricted to embryo-derived plasmatocytes or to lymph gland prohemocytes is sufficient to induce these phenotypes.

In addition we observed melanotic mass formation and lamellocyte differentiation upon expression of the dsRNA against *mRpS30 *or *cul4 *specifically in the fat body using the *FB-Gal4 *line (see Additional file 1, Figure S1 for its expression pattern). However, induction of melanotic tumors or lamellocytes was never observed for any of the five genes when dsRNA were expressed under the control of *MS1096*, *repo-Gal4*, *elav-Gal4 or cad-Gal4*, which respectively drive expression in the wing discs, the glial cells, the central nervous system and the gut. Thus gene knock down in the fat body, which plays a key role in innate immune response, can elicit a non-autonomous cellular immune response that culminates with the formation of melanotic nodules by the hemocytes. These results also show that the genes we tested act as melanotic tumor suppressors only in the immune system (blood cells and fat body) and not in other tissues, perhaps reflecting that only immune tissues can elicit melanotic capsule formation (see discussion).

All together, it appears that there are at least three different means of inducing melanotic mass formation: affecting the fat body, impinging on prohemocyte development or modifying differentiated blood cells.

### Embryo-derived plasmatocytes can cell autonomously differentiate into lamellocytes

The fact that some of the genes we recovered in the screen induced melanotic mass formation and lamellocyte differentiation when they were knocked-down specifically in embryonic plasmatocytes raised several questions. In particular we wondered whether these genes knock-downs provoked a cell-autonomous transformation of embryo-derived plasmatocytes into lamellocytes. Alternatively, these knock-downs might non-autonomously induce lamellocyte differentiation in the lymph gland. To test these possibilities, we monitored the fate of embryo-derived hemocytes that expressed the UAS-dsRNA as compared to that of the lymph gland-derived hemocytes. Accordingly, we used the flip-out technique to permanently label embryonic plasmatocytes and follow their fate in larval stages. Flies carrying the embryonic-specific plasmatocyte drivers *sn-Gal4 *or *gcm-Gal4 *recombined with a *UAS-FLP *were crossed to a strain bearing a flip-out cassette (*Act5C > FRT > CD2 > FRT > Gal4*) and a *UAS-GFP*. This technique allowed us to recover GFP-expressing circulating blood cells in third instar larvae in wild type conditions, whereas GFP-expressing cells were never observed in the absence of *UAS-FLP *(Figure 5A, B and D). Labeling with the plasmatocyte-specific marker P1/NimC1 confirmed that these cells were plasmatocytes (Additional file 3, Figure S3). Of note, probably due to the limited efficiency of the FLIP-FRT recombination, only a fraction of the blood cells was GFP^+^. Also, *sn-Gal4 *reproducibly gave higher frequency of GFP^+ ^cells than *gcm-Gal4*. Importantly, we never observed GFP^+ ^cells in the lymph glands demonstrating that these drivers are not sporadically expressed in this compartment during larval development and that embryo-derived plasmatocytes do not normally enter the lymph gland (Figure 5E-H).

We then made use of this technique to label the cells expressing a dsRNA targeting *ush*. As reported above (Table 2), expression of *ush *dsRNA under the control of *gcm-Gal4 *or *sn-Gal4 *was sufficient to induce the differentiation of lamellocytes in circulation, as revealed by *in situ *hybridization against α-*ps4 *and morphological analysis (Figure 5I and 5K). In the absence of Flipase, no GFP^+ ^cells were observed in circulation or in the lymph gland, indicating that these two drivers are not re-activated upon lamellocyte differentiation (Figure 5I, K, M and 5O). Strikingly, we observed GFP^+ ^lamellocytes in circulation in the presence of Flipase (Figure 5J and 5L) and these GFP^+ ^cells were also recovered in larvae with intact lymph glands. Therefore *ush *loss of function is sufficient to induce the cell autonomous transformation of embryo-derived plasmatocytes into lamellocytes. In addition, while *gcm-Gal4*-driven *ush *dsRNA induced lamellocyte production in circulation but not in the lymph gland (Figure 5J and 5N), we observed lamellocyte differentiation in both compartments using *sn-Gal4 *(Figure 5L and 5P). Again, none of the lamellocytes in the lymph gland were GFP^+^, indicating that they do not arise from *sn-Gal4*-expressing blood cells. This indicates that embryo-derived plasmatocytes participate in the production of lamellocytes both through cell autonomous and non-autonomous processes. Since *sn-Gal4 *gave rise to more GFP^+ ^cells than *gcm-Gal4*, it is possible that the non-autonomous induction of lamellocytes in the lymph gland is elicited in response to a threshold level of signalization by circulating hemocytes. To assess whether embryo-derived plasmatocytes also give rise to lamellocytes upon a natural immune challenge, we infected wild type larvae with eggs from the parasitoid wasp *L. boulardi*. As shown Figure 5Q-T, wasp infection induced lamellocyte differentiation and flip-out analysis showed that some of these lamellocytes are derived from cells that had expressed *gcm-Gal4 *or *sn-Gal4*. Thus, we conclude that embryo-derived plasmatocytes can differentiate into lamellocytes both upon intrinsic modification of the blood cell developmental program or in response to parasitoid infection.

**Figure 6:**
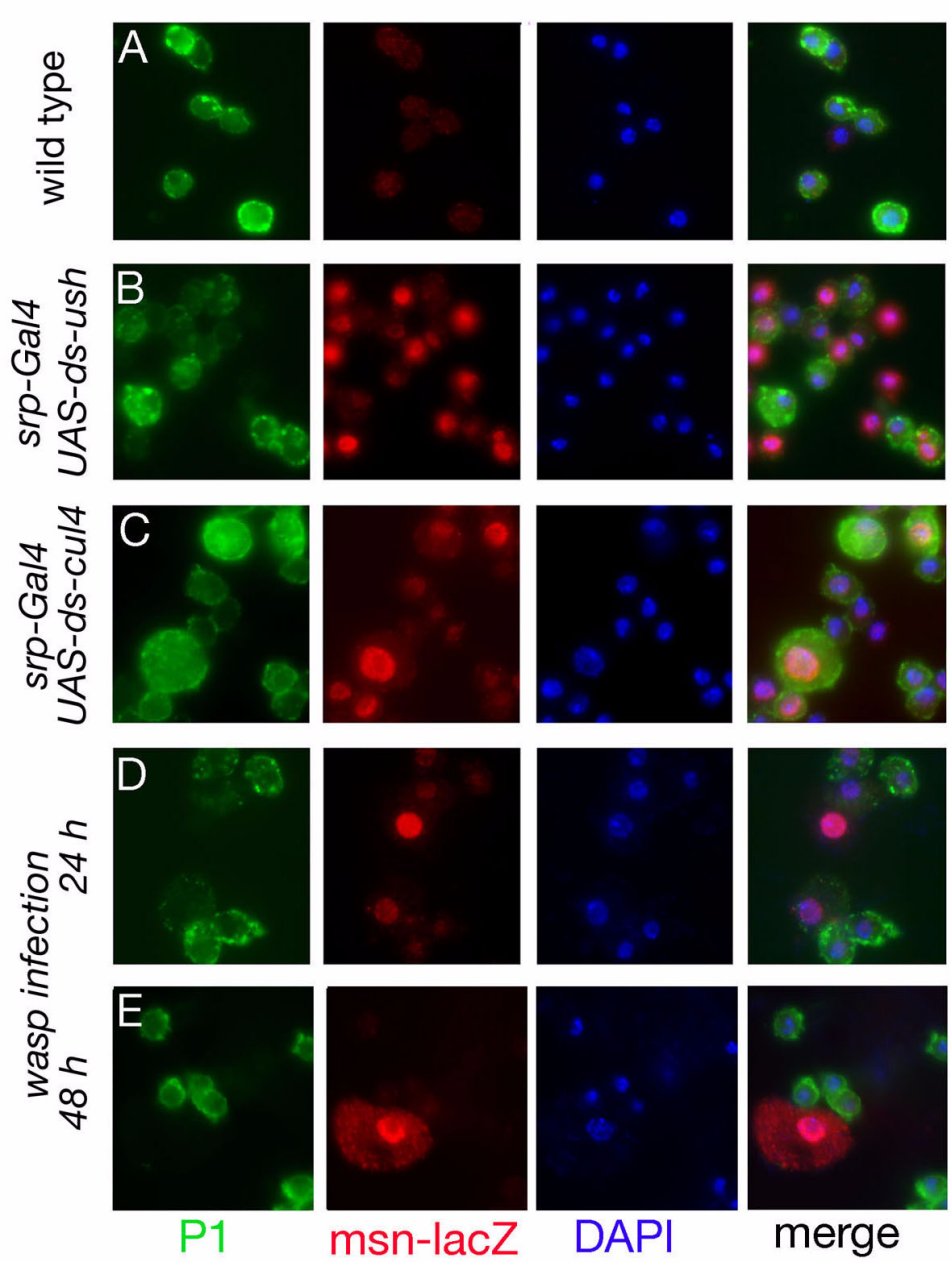
**Plasmatocyte and lamellocyte relationships**. (A-E) Double fluorescent immunostainings on blood smears from early third instar larvae showing the expression of the plasmatocyte specific marker P1 (green) and of the lamellocyte specific marker *msn-lacZ *(red). Nuclei were counterstained with DAPI (blue). (A) wild type larvae, (B-C) larvae expressing dsRNA against *ush *(B) or *cul4 *(C) under the control of *srp-Gal4*, (D-E): larvae infected by *L. boulardi*, 24 h (D) or 48 h (E) after parasitization.

To get further insights into the lamellocyte differentiation process induced by melanotic tumor suppressor genes or wasp infection, we analyzed circulating blood cell differentiation status by monitoring the expression of the plasmatocyte-specific marker P1/NimC1 and the lamellocyte-specific marker *msn-lacZ*. In control larvae, most blood cells expressed P1/NimC1 and only background β-gal staining was detected (Figure 6A). On the contrary, in third instar larvae expressing dsRNA against *ush *or *cul4 *under the control of *srp-Gal4*, *msn-lacZ *was expressed in most hemocytes (Figure 6B and 5C). Interestingly, a large fraction of these β-Gal^+ ^hemocytes also expressed P1, albeit often at lower levels. Similarly, 24 h after infection by *L. boulardi*, we found that most P1^+ ^cells also expressed *msn-lacZ *(Figure 6D). However, cells expressing both markers were rare 48 h after infestation: almost all the β-Gal^+ ^cells corresponded to typical lamellocytes with large flattened morphology, big nuclei and no P1 staining, whereas P1^+ ^hemocytes had the characteristic small and round morphology of plasmatocytes (Figure 6E). Thus, together with the above results, these data suggest that lamellocyte differentiate from plasmatocytes *via *a stepwise process implicating activation of *msn-lacZ*, change in cell morphology and repression of P1 expression.

## Discussion

In this study we have conducted a loss of function screen to identify factors that regulate Drosophila blood cell development and/or function. Thus far, screens aiming at uncovering genes controlling hemocyte development relied primarily on the use of zygotic mutants potentially acting in other tissues ([25] and references therein) or on misexpression of factors potentially not expressed in blood cells [55,56]. Likewise, the conclusion that a given melanotic suppressor gene was specifically affecting blood cells rather than an other tissue was based mostly on the targeted expression of dominant negative (*e.g*. *ush *or *lwr *with *cg-Gal4*) [41,57], rescue experiments (*e.g*. *vsp35 *with *hml-Gal4*, or *ADGF-A *with *cg-Gal4*) [58,59], or other indirect evidences (*e.g*. *cactus*, *yantar, zfrp8*) [40,60,61]. To target more specifically genes expressed in hemocytes and to be able to study genes required for embryonic or early larval viability, we choose a tissue-specific loss of function approach that relied on the use of a collection of *UAS-dsRNA *transgenic lines and of three different Gal4 drivers expressed in the hemocytes. To the best of our knowledge this is the first time that such cell-targeted loss of function approach is used to identify new regulators of blood cell function and development.

By screening 1340 genes by RNA interference, we recovered 96 candidate melanotic tumor suppressor genes among which 59 were confirmed with secondary RNAi lines and/or by genetic means. This corresponds to a hit rate of 7.1% (4.4% if we only consider confirmed hits). For comparison, a recent genome wide RNAi screen for genes involved in intestinal pathogenic bacterial infection resulted in 8.6% of hits [62], while a gain of function screen in larval hemocytes led to 3.2% of hits [55]. Yet, in neither case a systematic validation of the candidates was carried out. In a genome-wide RNAi screen for genes affecting adult thorax development (19.6% of hits, excluding genes required for viability) [63], 63% of the 73 candidates retested with secondary RNAi lines were confirmed, which is similar to our confirmation rate (94 candidates retested with secondary RNAi lines, 62% confirmed). Since in 31% of the cases, the primary and secondary RNAi lines targeted overlapping sequences, we cannot rule out that some of the melanotic masses might be caused by off-target effects. The analysis of the expression pattern of the candidates and of the RNAi efficiency might help resolving this issue. Interestingly though, data mining showed that most of the confirmed genes are functionally connected to one another or to a previously identified melanotic suppressor, further substantiating our conclusion that they work in a common process. Thus, altogether, this screen allowed us to find 59 genes that likely contribute to larval blood cell homeostasis, among which 55 had not been directly associated with blood cell development or function before.

In contrast to the customary partition of the melanotic tumor suppressor into class I/ class II genes [25,27], we propose that the capacity to elicit melanotic mass development is restricted to the blood cells and the fat body (*i.e*. immune tissues). Indeed, none of the candidates we tested induced lamellocytes or melanotic masses when knocked-down in other territories. Similarly, knocking down the melanotic tumor suppressors *ddb1 *or *DREF *by RNAi in several non-immune tissues did not induce melanotic masses [42,50], and Hop^Tuml ^or a dominant negative form of GCM induced melanotic masses when ectopically expressed respectively in blood cells or in the fat body, but not in other tissues [31,64,65]. All together, there is no strong evidence that melanotic capsule can arise from genetic defects outside the hematopoietic system or the fat body. Indeed, the rare "class I" mutations that have been studied (*kurtz*, *tuW *and *tu-Sz^ts^*) have been shown to affect fat body integrity [28,66]. While our results demonstrate for the first time that fat body-specific loss of function can cause lamellocyte differentiation and melanotic mass production, the mechanisms involved remain unclear. The fat body may be a specific source of signaling molecules that activate lamellocyte differentiation or more generally control hemocyte differentiation. Conversely, several lines of evidence show that hemocytes can signal to the fat body to regulate the humoral immune response [12,13,67,68]. This cross talk between the fat body and the hematopoietic system is likely to play a crucial role in coordinating the cellular and humoral immune response to ensure efficient defense of the organism.

Our results show that, within the hematopoietic system, loss of function in larval lymph gland prohemocytes, in differentiated larval blood cells or in embryonic plasmatocytes is sufficient to induce melanotic masses in the larvae. The only resilient blood cell type seemed to be the crystal cells, as we never observed nodules or lamellocytes induction with the *lz-Gal4 *driver, even by overexpressing with this driver the two paradigmatic melanotic tumor inducers Toll^10b ^and Hop^Tum ^(AAR, unpublished observations). Thus melanotic tumor formation can serve as a read out to identify genes potentially controlling several steps of blood cell development. For instance, it may help defining the gene regulatory network that control the maintenance of a pool of stem-like blood cells in the lymph gland cortical zone [8,9]. Actually, the respiratory chain component ND75 that we recovered in the screen, was recently shown to participate in the maintenance of these progenitors by controlling the levels of reactive oxygen species [39].

An important finding that stems out of the analysis of some genes identified in the screen is that impinging on the function of embryo-derived hemocytes is sufficient to cause lamellocyte differentiation in the larvae. Our cell lineage analysis demonstrates that embryo-derived plasmatocytes cell-autonomously give rise to lamellocytes in response to a genetic defect (*ush *loss) or to wasp infection. These results are consistent with and extend the recent observation that embryo-derived hemocytes can differentiate in lamellocytes after wasp infection [24]. Whereas Markus *et al*. proposed that lamellocytes differentiate from hemocyte precursors [24], our analysis strongly suggests that they derive from plasmatocytes through a step-wise process. In addition, we found that the presence of "mutant" embryo-derived blood cells induced non-autonomously the differentiation of lamellocytes in the lymph gland, indicating that circulating hemocytes signal to this hematopoietic organ. Mutations in the EBF transcription factor Collier (which is expressed in the posterior signaling centre) or in the JAK/STAT signaling pathway (which is active in the medullary zone) were shown to induce precocious differentiation of the lymph gland progenitors and to suppress lamellocyte fate, strongly suggesting that lamellocyte differentiate solely in the lymph gland from a pool of progenitors [8,22]. Alternatively, we propose that the posterior signaling centre may orchestrate differentiation into lamellocytes of both circulating and lymph gland blood cells. Thereby, full blown lamellocyte differentiation and melanotic nodule formation would result from a cross talk between the patrolling larval blood cells and the lymph gland.

Finally our result shed new light on the function of the Friend of GATA transcription cofactor Ush, which had already been implicated in several steps of blood cell development [41,69-72]. Consistent with our results, it was shown that hypomorphic *ush *zygotic mutations or the ectopic expression of a dominant negative form of Ush under the control of *cg-Gal4 *induced lamellocyte differentiation [41]. Moreover, it was proposed that Ush was required in the lymph gland to prevent lamellocyte differentiation in a putative plamatocyte/lamellocyte common progenitor [41,69]. Yet the fate of the *ush *mutant cells had not been tracked. Remarkably, we demonstrate here that *ush *function is not restricted to the lymph gland. Indeed *ush *loss in circulating plasmatocytes during embryogenesis is sufficient to cause the cell-autonomous transformation of these cells into lamellocytes and to promote lamellocyte development in the lymph gland. Thus down-regulation of *ush *function in the circulating larval blood cells could be an initiating event in the immune response that leads to melanotic mass formation. Our results also identify *ush *as the first gene controlling the fate of the circulating larval blood cells and lay the basis for the analysis of the gene networks controlling this hematopoietic compartment.

## Conclusions

In this study, we show that lamellocyte differentiation and melanotic tumor formation can be elicited specifically by defects in different immune compartments (embryo-derived blood cells, larval hematopoietic organ or fat body). These results shed new lights on the coordination of the cellular immune response and on blood cell lineage relationships in Drosophila. Notably, we demonstrate that embryo-derived plasmatocytes are plastic cells that can differentiate into lamellocytes and that *ush *is a key regulator of this process. Finally, this work pinpoints several new genes and pathways controlling Drosophila blood cell homeostasis. Their identification paves the way for future experiments aiming at dissecting their mechanism of action and their interplay with other known key regulators of Drosophila hematopoiesis. It is anticipated that deciphering the function of these genes in the different blood cell types will shed new light on the mechanisms controlling blood cell homeostasis and cellular immune response in Drosophila and, by homology, in mammals.

## Methods

### Fly strains and genetic crosses

Flies were raised at 25° on standard cornmeal and agar media. The following strains were used: *srp-Gal4 *[73]; *hml*Δ-*Gal4 *[37]; *cg-Gal4 *[36]; *gcm-Gal4 *[35]; *sn-Gal4 *(Zanet *et al.*, in preparation); *fb-Gal4 *(from M. Meister); *cad-Gal4*, *tepIV-gal4 *(from Kyoto DGRC); *MS1096, en-Gal4, repo-Gal4, elav-Gal4*, *Act5C *> *FRT > CD2 > FRT > Gal4, UAS-FLP, msn-lacZ, UAS-EGFP, UAS-mCD8GFP *(from Bloomington). *UAS-dsRNA *transgenic lines were obtained from the Japanese National Institute of Genetic (NIG), the Vienna Drosophila Resource Center (VDRC) and Bloomington.

A collection of 1941 *UAS-dsRNA *transgenic lines targeting 1341 genes (Additional file 4, Table S1) obtained from NIG was analyzed in our primary screen. For this, 5-7 virgin females carrying either the *hml*Δ-*Gal4 *or the *srp-Gal4 *driver were crossed to 3-4 males carrying the different *UAS-dsRNA *transgenes. Vials were changed every two days and presence of melanotic masses was evaluated in the progeny by examinating under the dissection microscope an average of 20 wandering larvae. Candidate *UAS-dsRNA *lines inducing melanization with at least one driver were systematically retested with both drivers as well as with *cg-Gal4 *and the corresponding "tumor" indices (percentage of larvae harboring at least one melanotic masse) were determined on a minimum of 50 larvae. For validation of the candidate genes, independent secondary *UAS-dsRNA *lines or mutant alleles were obtained from VDRC and Bloomington.

### Hemocyte labeling

To observe the circulating larval hemocytes, third instar larvae were thoroughly washed in PBS and ethanol 75% and bled on polylysine-coated glass slides (Nunc). For immunostaining and/or phalloidin labeling, hemocytes were briefly air-dried and then fixed for 15 min in 4% paraformaldehyde in PBS. After two washes in PBS, hemocytes were permeabilized for 15 min in PBS, 0.3% Triton (PBST), rinsed twice in PBST-1% BSA, and blocked 15 min in PBST-1% BSA. Hemocytes were incubated 2 h at room temperature or overnight at 4°C with primary antibody. After several 15 min washes, cells were incubated 2 h with secondary antibody and / or with phalloidin SR101 (1:200) (SIGMA) and washed 4 times in PBST. Slides were mounted in Vectashield-DAPI medium. Double fluorescent immuno-staining and *in situ *hybridization on circulating blood cells were performed as described in [74].

For lymph gland analysis, third instar larvae were dissected in ice cold PBS, fixed for15 min in 4% paraformaldehyde, washed three times 15 min in PBST and pre-incubated for 1 h at 60°C in Hybridization Buffer (HB: 50% formamide, 2 × SSC, 1 mg/ml Torula RNA, 0.05 mg/ml Heparin, 2% Roche blocking reagent, 0.1% CHAPS, 5 mM EDTA, 0.1% Tween 20). Larvae were then incubated overnight at 60°C with DIG-labeled RNA probe, washed twice for 1 h in 50% HB-50% PBST at 60°C and three times 20 min in PBST-1% BSA at room temperature, before 3 h incubation with sheep anti-DIG antibody conjugated to alkaline phosphatase (1:2000; Roche). Finally, larvae were extensively washed in PBST and the *in situ *hybridization signal was revealed with FastRed or NBT/BCIP substrates. Lymph glands were mounted in 50% glycerol-PBS or in Vectashield-DAPI.

The following primary antibodies were used: mouse anti-P1/NimC1 (1:30) (kind gifts from I. Ando, [75]), rabbit anti-ϐ galactosidase (1:1000, Cappel), rabbit anti-GFP (1:1000; Torrey). The corresponding secondary antibodies coupled to Alexa fluor 488 or 555 (1:400) (Molecular Probes). For in *in situ *hybridization, we used DIG-UTP labeled α-*ps4 *and *tepIV *anti-sense RNA probes [53,76]. For *in situ *hybridization followed by immunostaining, primary antibodies were used 5 times more concentrated.

### Wasp egg parasitization

To obtain synchronous larval populations, females were left to lay eggs for 4-6 hours. Second instar larvae were submitted to infection by *L. boulardi *during 2-4 h, and then allowed to develop 24 or 48 h before being analyzed as described above.

## Authors' contributions

AAR and KB performed most experiments, contributed to acquisition of all data, analyzed and interpreted the data. CP participated in the design of the experiments and interpretation of the results. CP, VG, DO, BA, FR participated in the realization of the screen and provided important genetic tools. JZ provided important genetic tools. MH and LW conceived and coordinated the project. AAR, KB, CP, FR and MH have been involved in drafting the manuscript. LW wrote the manuscript. All authors read and approved the final manuscript.

## Supplementary Material

Additional file 1 **Figure S1**. Expression pattern of different Gal4 lines in the embryo and in the circulating hemocytes, lymph gland and fat body of third instar larvae. The activity of the indicated Gal4 lines was revealed using either a *UAS-lacZ *or a *UAS-GFP *reporter transgene whose expression was detected respectively by *in situ *hybridization against *lacZ *in embryos or by fluorescent immunostaining against GFP in third instar larvae. Circulating hemocyte actin cytoskeleton was labeled with phalloidin (red). Lymph gland and fat body nuclei were counterstained with DAPI (blue). Click here for file

Additional file 4 **Table S1**. Results from the primary and secondary screens. Click here for file

Additional file 5 **Table S2**. Validation of candidates with secondary *UAS-dsRNA *lines. Click here for file

Additional file 6 **Table S3**. List of confirmed melanotic suppressor genes. Click here for file

Additional file 2 **Figure S2**. High magnification view of a melanotic mass (induced using *srp-Gal4, UAS-GFP; UAS-ds-Ush*). GFP and phalloidin staining show that the mass is surrounded by lamellocytes. Click here for file

Additional file 3 **Figure S3**. Flip-out analysis of the expression pattern of *sn-Gal *and *gcm-Gal4 *in circulating larval blood cells. Blood cells smears from third instar larvae of the indicated genotypes were processed to reveal nuclear-GFP (in green) and P1/NimC1 (in red) expression by double fluorescent immuno-labeling. Nuclei were counterstained with DAPI (blue). Click here for file
