# Supplementary figures and images for: An in vivo RNA interference screen identifies gene networks controlling Drosophila melanogaster blood cell homeostasis
Source: BMC Dev Biol. 2010 Jun 11;10:65. doi: 10.1186/1471-213X-10-65 (PMC2891661; doi:10.1186/1471-213X-10-65)

Additional Figure 1

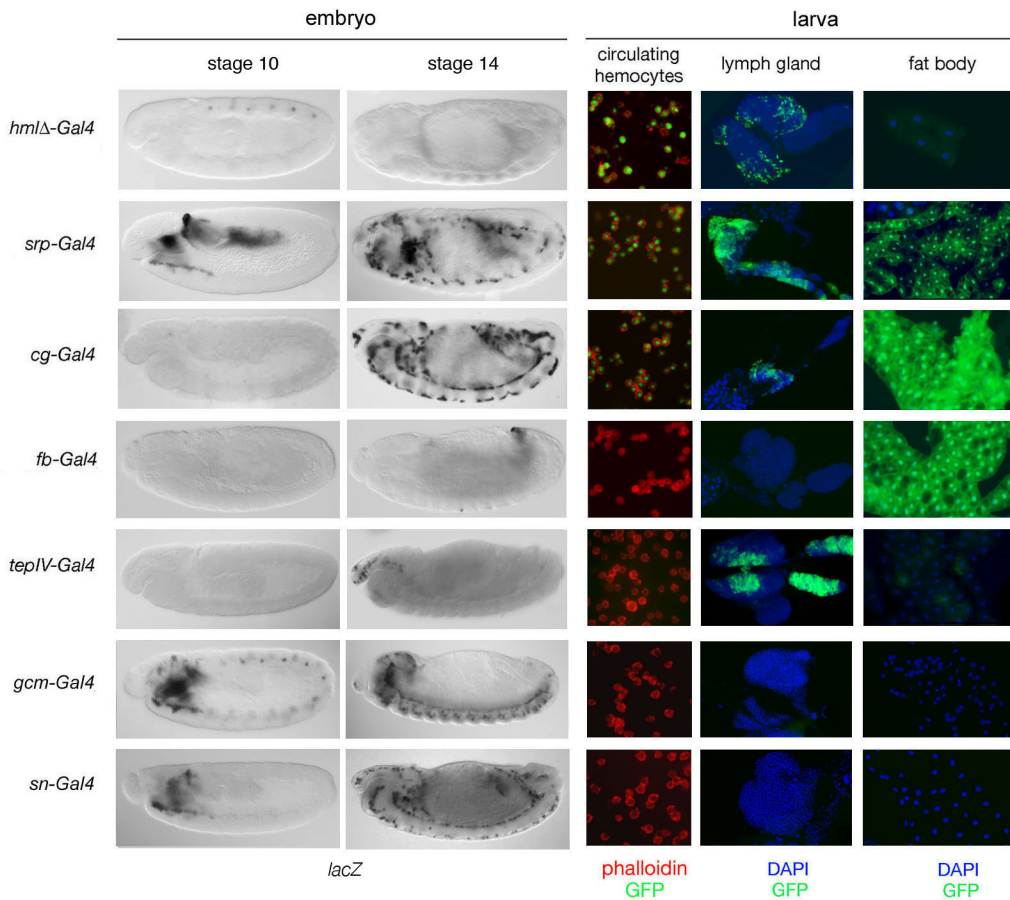

Supplement: Additional file 1 — Figure S1. Expression pattern of different Gal4 lines in the embryo and in the circulating hemocytes, lymph gland and fat body of third instar larvae. The activity of the indicated Gal4 lines was revealed using either a UAS-lacZ or a UAS-GFP reporter transgene whose expression was detected respectively by in situ hybridization against lacZ in embryos or by fluorescent immunostaining against GFP in third instar larvae. Circulating hemocyte actin cytoskeleton was labeled with phalloidin (red). Lymph gland and fat body nuclei were counterstained with DAPI (blue). [file 1471-213X-10-65-S1.PDF]

## Additional Figure 3

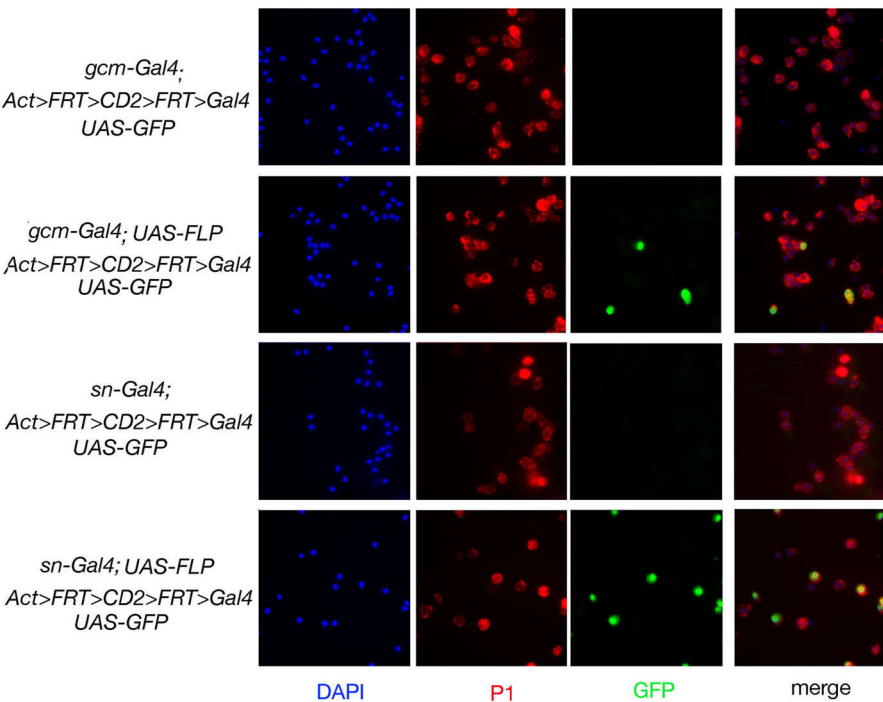

Supplement: Additional file 5 — Table S2. Validation of candidates with secondary UAS-dsRNA lines. [file 1471-213X-10-65-S5.PDF]

# Additional Figure 2

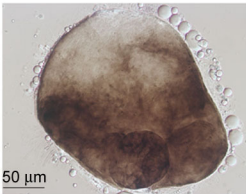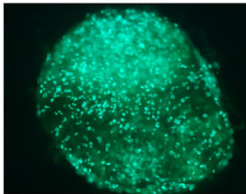

GFP

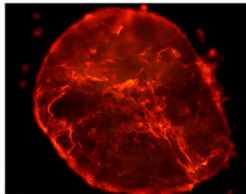

phalloidin

Supplement: Additional file 2 — Figure S2. High magnification view of a melanotic mass (induced using srp-Gal4, UAS-GFP; UAS-ds-Ush). GFP and phalloidin staining show that the mass is surrounded by lamellocytes. [file 1471-213X-10-65-S2.PDF]
