# Supplementary material for: An in vivo RNA interference screen identifies gene networks controlling Drosophila melanogaster blood cell homeostasis
Source: BMC Dev Biol. 2010 Jun 11;10:65. doi: 10.1186/1471-213X-10-65 (PMC2891661; doi:10.1186/1471-213X-10-65)
Supplement: Additional file 4 — Table S1. Results from the primary and secondary screens. [file 1471-213X-10-65-S4.PDF]

Additional Table S2

Validation of candidates with secondary *UAS-dsRNA* lines

		primary UAS-dsRNA					secondary UAS-dsRNA					primary / secondary
CG	SYMBOL	S19	OTE	*srp-Gal4*	*hml*Δ*-Gal4*	*cg-Gal4*	S19	OTE	*srp-Gal4*	*hml*Δ*-Gal4*	*cg-Gal4*	
CG6311	Edc3	0.7	>20	20.5	4	100	0.97	3	0	0	0	NO
CG12276	*Aos1*	1	0	0	10	56.4	1	0	1.9	21.6	22.9	OV
CG31256	*Brf*	1	0	15.6	3.6	50.7	1	2	14.7	2	34.5	NO
CG12653	*btd*	0.34	>20	37.3	0	leth.	0.94	8	2.5	0	0	NO
CG5848	*cact*	0.98	4	12	4.4	25.4	n.a.	n.a.	n.a.	n.a.	n.a.	n.a.
CG10362	CG10362	1	0	6	0	3.7	1	0	1.2	0	0	NO
CG11837	CG11837	1	0	5	0	84.1	1	0	0	0	11.7	NO
CG12661	CG12661	1	1	2.5	9	54.5	1	1	0	0	0	OV
CG12772	CG12772	0.55	>20	14	0	75	1	1	0	0	0	NO
CG12870	CG34362	0.34	>20	30	0	65.2	1	0	2.7	0	5.5	NO
CG14100	CG14100	1	0	8	2.4	3.6	1	0	0	0	0	OV
CG14230	CG14230	1	0	5	2.9	70.7	0.99	2	4.2	4.7	72	NO
CG14512	CG14512	1	0	5.6	0	13.2	1	0	4	0	22.8	OV
CG15247	CG15247	0.6	>20	1.4	3.6	13.3	0.96	8	0	0	0	OV
CG15314	CG15314	0.84	14	12.3	1.9	leth.	1	0	0	1.6	2	OV

CG15347	CG15347	1	0	0	0	11.1	1	0	0	0	0	OV
CG15784	CG15784	0.93	10	7.5	6	0	0.92	7	15.4	5.5	2.9	OV
CG17331	CG17331	1	0	40	4	leth.	1	0	70.5	9.2	leth.	OV
CG17646	CG17646	0.99	1	0	0	53.2	1	0	0	0	0	NO
CG1832	CG1832	0.84	>20	21	7.7	9.3	1	1	0	0	0	NO
CG1924	CG1924	0.63	2	8	0	3.2	0.95	2	0	0	0	NO
CG2010	CG2010	0.95	>20	5.6	0	5.9	1	0	0	1.9	0	OV
CG30156	CG30156	0.99	2	3.5	0	57.7	1	0	0	1.9	1.7	OV
CG31044	CG31044	0.66	>20	13.6	0	1.8	0.15	465	45.9	0	68.2	OV
CG5086	CG32767	0.42	>20	40	1.8	leth.	0.88	20	2.4	2.6	0	NO
CG4407	CG4407	1	0	30	2	19	1	0	8.6	5	52.3	NO
CG4707	CG4707	1	1	0	1.2	9.7	1	0	0	1.8	0	OV
CG5222	CG5222	1	0	3.9	0	18.5	1	1	11.5	0	0	NO
CG6197	CG6197	1	0	45	0	leth.	1	0	31.3	6	100	NO
CG6322	CG6322	1	0	4.5	5	37.1	1	0	50	3.3	leth.	NO
CG7033	CG7033	1	2	39	1.4	2	0.98	3	60.8	0	84.4	NO
CG7757	CG7757	0.99	1	50.8	6	leth.	1	0	32	5.4	58.8	NO
CG7845	CG7845	1	1	3.6	0	48.6	1	0	11.6	5.4	leth.	NO
CG8444	CG8444	1	2	3	0	10	0.99	1	2	0	13.5	OV

CG8878	CG8878	1	1	12.2	0	3.9	1	0	0	0	0	NO
CG9107	CG9107	1	0	7.3	2.5	8.8	1	0	29.6	3.5	18.3	NO
CG9305	CG9305	0.94	11	13.9	7.7	leth.	0.99	1	18	3.1	5.1	NO
CG9663	CG9663	1	0	31	0	0.9	n.a.	n.a.	n.a.	n.a.	n.a.	n.a.
CG9853	CG9853	1	1	8.4	0	23.1	1	1	1.9	2	2.1	OV
CG15349	*Cp7Fa*	0.95	13	10.3	0	leth.	0.94	13	17.7	0	25	OV
CG3889	*CSN1b*	1	0	10.3	3.6	3.8	1	1	6	4.9	3.6	NO
CG8711	*cul-4*	1	2	46	39.4	77.1	1	0	32.1	0	91.4	NO
CG6292	*CycT*	1	0	33.3	0	10	1	0	3.4	0	2.1	OV
CG9745	*D1*	0.99	1	0	0	26.5	1	0	0	3.7	0	NO
CG2048	*dco*	0.94	4	8.9	0	51.4	0.91	15	0	0	0	NO
CG5838	*Dref*	1	0	13.6	2.5	100	1	0	17.4	15.4	100	OV
CG10846	*dyn-p25*	1	1	2.8	5	0	1	1	0	0	0	OV
CG15410	*E23*	0.3	18	13.1	11.4	27.6	0.99	1	0	0	0	NO
CG11901	*Ef1gamma*	1	1	17.2	0	leth.	0.99	2	95.2	0	17	NO
CG1873	*Ef1alpha100E*	0.98	1	9.5	2.5	18.4	0.98	1	5.4	0	10	OV
CG5605	*eRF1*	1	1	15.9	0	16.7	1	1	32	0	leth.	NO
CG10033	*for*	0.94	2	0	0	16.5	1	0	20.5	4.3	leth.	NO
CG4396	*fne*	1	0	25	1.5	10.8	1	0	3.2	2	20	OV

CG2522	*Gtp-bp*	1	0	18	21.4	leth.	1	0	0	0	85.7	NO
CG31359	*Hsp70Bb*	0.25	8	7.1	7.6	leth.	0.75	3	0	0	0	NO
CG6489	*Hsp70Bc*	0.2	9	26.4	15.5	68.1	0.67	5	27.9	7.5	13.9	NO
CG11990	*hyx*	1	0	55	8.3	7.7	1	0	63.8	18.8	39.1	NO
CG18039	*KaiRIA*	1	0	2	8	6.8	0.97	2	0	0	0.9	NO
CG5649	*kin17*	1	1	30	1.3	1.3	1	1	0	2.7	1.8	OV
CG1994	*l(1)G0020*	1	1	12	0	17.5	0.99	1	0	0	21.8	NO
CG11295	*l(2)dtl*	1	0	4	11.3	23.6	0.98	5	0	5	7.7	NO
CG3354	*Mst77F*	1	0	0	4.7	26.9	0.72	1	3.4	0	0	OV
CG16973	*msn*	0.97	8	4	0	31.6	1	0	0	0	0	NO
CG10603	*mRpL13*	0.79	1	4	12	36	1	0	5.5	32.5	100	OV
CG12954	*mRpL41*	1	1	0	0	83.6	1	1	5.4	8.4	97.6	OV
CG1577	*mRpL52*	1	0	1	5.2	86.8	1	0	2.6	0	100	OV
CG8470	*mRpS30*	1	2	0	7.1	94.3	1	0	5.8	8.2	97.8	NO
CG8025	*Mtr3*	1	2	12.5	0	21.7	1	0	11.5	3.7	41.7	OV
CG2286	*ND75*	1	0	8	5.2	92.9	1	0	4.3	16.3	76.7	NO
CG1857	*nec*	0.76	14	21.5	17.5	leth.	0.37	169	26.6	14.6	leth.	OV
CG12154	*oc*	0.7	>20	67	9.4	39.4	1	0	0	0	0	NO
CG10295	*Pak*	1	0	24	0	leth.	1	0	4	0	0	NO

CG5786	*ppan*	1	1	31.3	16	45.7	1	1	10.9	0	4.4	NO
CG18495	*CG18495*	1	0	26.7	4.8	leth.	0.98	3	1.6	0	0	OV
CG4904	*Pros35*	1	0	2.8	5	leth.	1	0	5.1	1.4	leth.	NO
CG5519	*Prp19*	1	0	9.3	0	0	0.98	1	35.3	0	22.7	NO
CG9441	*Pu*	0.98	4	3.2	0	22	0.99	1	0	0	0	OV
CG13194	*pyr*	0.49	>20	28	0	1.8	0.11	220	22.9	5.2	0	NO
CG14999	*RfC4*	1	0	25.9	0	23	1	1	0	8.6	0	NO
CG5371	*RnrL*	1	0	0	0.9	19.8	1	0	3.9	0	5.6	NO
CG6846	*RpL26*	1	0	14.3	0	leth.	1	0	leth.	7.8	leth.	OV
CG11522	*RpL6*	1	0	4.8	6.7	0	1	1	leth.	5	leth.	NO
CG7014	*RpS5b*	1	0	8.2	3.6	0	0.99	1	20	3.9	10	OV
CG7885	*RpII33*	1	0	0	10	95	1	0	8	13.6	34.5	NO
CG11888	*Rpn2*	1	0	5	4	leth.	1	0	36.1	1.3	47.6	NO
CG4659	*Srp54k*	1	0	31.7	0	leth.	1	0	26.4	3.6	leth.	NO
CG11941	*skpC*	0.71	4	0	0	10.3	0.78	3	7.5	0	14.3	OV
CG12225	*Spt6*	0.99	2	32.1	1.4	41.4	1	0	92	82.7	leth.	NO
CG32211	*Taf6*	1	0	34.2	3	10.8	1	0	0	0	22.4	NO
CG11527	*Tig*	1	1	0	0	12.1	0.99	2	2.9	0	10	NO
CG6121	*Tip60*	1	0	14.3	0	1.8	1	0	0	0	0	OV

CG8595	*Toll-7*	1	2	8.3	5	50	1	0	0	0	0	OV
CG10117	*ttv*	1	0	1.7	0	15.6	1	0	0	0	0	NO
CG2762	*ush*	1	1	40	17	67.7	0.96	7	37	48.1	75.5	NO
CG17437	*wds*	1	0	0	0	52.4	1	0	4	0	43.4	NO
CG9433	*Xpd*	1	0	0	0	12.3	1	0	0	1.9	0	NO

Tumor indices (%) are indicated for each driver. S19: specificty score (total number of 19-mer/(number of off -target + number of on-target of each 19mer)). OTE: number of off-targets. Leth.: lethal. OV: overlapping dsRNA. NO: non-overlapping dsRNA. N.a.: non-available
