# Supplementary material for: An in vivo RNA interference screen identifies gene networks controlling Drosophila melanogaster blood cell homeostasis
Source: BMC Dev Biol. 2010 Jun 11;10:65. doi: 10.1186/1471-213X-10-65 (PMC2891661; doi:10.1186/1471-213X-10-65)
Supplement: Additional file 6 — Table S3. List of confirmed melanotic suppressor genes. [file 1471-213X-10-65-S6.PDF]

Additional Table S3

List of confirmed melanotic suppressor genes

CG	NAME	SYMBOL	inferred function	other information
CG12276	*Aos1*	*Aos1*	Ubiquitin activating enzyme; Toll signaling	zygotic mutation associated to melanotic masses and lamellocytes *(*$Aos1^{c06048}$*)*
CG14512	*Asparagine-linked glycosylation 13*	*Alg13*	UDP-GlcNAc transferase; protein sorting	
CG8444	*ATP6AP2*	*ATP6AP2*	vacuolar H(+)-ATPase, putative receptor; signaling	
CG9305	*B double prime 1*	*Bdp1*	TFIIIB subunit; transcription	
CG31256	*Brf*	*Brf*	TFIIIB subunit; transcription	
CG5848	*cactus*	*cact*	inhibitor of NF-kB transcription factors; Toll signalling, immune response	known as melanotic tumor tumor suppressor [32]
CG7033	*Cct2*	*Cct2*	chaperonin-containing T-complex; protein folding	
CG15784	CG15784	CG15784	unknown	
CG31044	CG31044	CG31044	unknown	
CG7845	CG7845	CG7845	putative pre-ribosomal particle constituent; ribosome assembly?	
CG15349	*Chorion protein a at 7F*	*Cp7Fa*	unknown	
CG3889	*COP9 complex homolog subunit 1 b*	*CSN1b*	COP9 signalosome; protein degradation	partner of known melanotic tumor suppressors (*CSN5* [43]; *CSN8* [44])
CG8711	*cullin-4*	*cul-4*	ubiquitin-protein ligase; protein degradation, transcription, DNA repair	partner of known melanotic tumor suppressor (*Ddb1* [47])

CG11837	*Dimethyladenosine transferase 1*	*Dim1*	ribosomal RNA processing	
CG5838	*DNA replication-related element factor*	*Dref*	transcription factor; transcription	known as melanotic tumor suppressor [39]
CG11901	*Ef1g*	*Ef1g*	translation elongation factor; translation	
CG1873	*Elongation factor 1a100E*	*Ef1a100E*	translation elongation factor; translation	partner of a known melanotic tumor suppressor (*Hsc70-4* [45])
CG5605	*eukaryotic release factor 1*	*eRF1*	translation termination factor; translation	
CG4407	*Flavin adenine dinucleotide synthetase 1*	*Fad1*	mitochondrial respiratory chain; ATP synthesis	
CG10033	*foraging*	*for*	cGMP-dependent kinase; signaling	
CG4396	*found in neurons*	*fne*	RNA splicing	
CG2522	*GTP-binding protein*	*Gtp-bp*	signal recognition particle receptor; protein targeting	
CG6489	*Heat-shock-protein-70Bc*	*Hsp70Bc*	chaperon; protein folding	partner of a known melanotic tumor suppressor (*Hsc70-4* [45])
CG11990	*hyrax*	*hyx*	Paf1 complex component; elongation of transcription	
CG5222	*Ints9*	*Ints9*	integrator complex subunit; transcription, RNA processing	
CG1994	*lethal (1) G0020*	*l(1)G0020*	N-acetyltransferase; ribosomal constituent processing	
CG11295	*lethal-(2)-denticleless*	*l(2)dtl*	response to DNA damage; DNA replication	partner of a known melanotic tumor suppressor (*Ddb1* [47])
CG10603	*mitochondrial ribosomal protein L13*	*mRpL13*	mitochondrial large ribosomal subunit; translation	partner of a known melanotic tumor suppressor (*mRpL55* [42])
CG12954	*mitochondrial ribosomal protein L41*	*mRpL41*	mitochondrial large ribosomal subunit; translation	partner of a known melanotic tumor suppressor (*mRpL55* [42])

CG1577	*mitochondrial ribosomal protein L52*	*mRpL52*	mitochondrial large ribosomal subunit; translation	partner of a known melanotic tumor suppressor (*mRpL55* [42])
CG8470	*mitochondrial ribosomal protein S30*	*mRpS30*	mitochondrial small ribosomal subunit; translation	partner of a known melanotic tumor suppressor (*mRpL55* [42])
CG8025	*Mtr3*	*Mtr3*	exosome component; RNA processing	
CG2286	*NADH:ubiquinone reductase 75kD subunit precursor*	*ND75*	mitochondrial respiratory chain complex; ATP synthesis	lymph-gland driven dsRNA induces lamellocyte formation [37]
CG1857	*necrotic*	*nec*	serine-type endopeptidase inhibitor; Toll signalling, immune response	
CG14230	*Nucleolar protein 8*	*Nol8*	ribosomal RNA processing	
CG5786	*peter pan*	*ppan*	pre-ribosomal particles constituent; ribosome assembly	
CG4904	*Proteasome 35kD subunit*	*Pros35*	proteasome component; protein degradation	
CG17331	*Proteasome subunit, beta type, 2*	*Psmb2*	proteasome component; protein degradation	
CG5519	*Prp19 (GTP-binding-protein)*	*Prp19 (Gbp)*	RNA splicing	
CG7757	*Prp3*	*Prp3*	RNA splicing	
CG6322	*Prp4*	*Prp4*	RNA splicing	
CG13194	*pyramus*	*pyr*	cytokine; fibroblast growth factor receptor signaling	zygotic mutation associated to melanotic masses and lamellocytes (*$pyr^{MB02808}$*)
CG14999	*Replication factor C subunit 4*	*RfC4*	replication factor C component; DNA replication	partner of known melanotic tumor suppressor (*RfC1* [46]); zygotic mutations associated to melanotic masses and lamellocytes (*$RfC4^{B6}$:$RfC4^{B6}$/$RfC4^{A18}$*)
CG5371	*Ribonucleoside diphosphate reductase large subunit*	*RnrL*	ribonucleoside-diphosphate reductase complex; DNA replication	

CG6846	*Ribosomal protein L26*	*RpL26*	cytosolic large ribosomal subunit; translation	partner of a known melanotic tumor suppressor (*RpS6* [41])
CG11522	*Ribosomal protein L6*	*RpL6*	cytosolic large ribosomal subunit; translation	partner of a known melanotic tumor suppressor (*RpS6* [41])
CG7014	*Ribosomal protein S5b*	*RpS5b*	cytosolic small ribosomal subunit; translation	partner of a known melanotic tumor suppressor (*RpS6* [41])
CG7885	*RNA polymerase II 33kD subunit*	*RpII33*	RNA polymerase II subunit; transcription	
CG11888	*Rpn2*	*Rpn2*	proteasome component; protein degradation	
CG9107	*Rrp7*	*Rrp7*	ribosomal RNA processing	
CG4659	*Signal recognition particle protein 54k*	*Srp54k*	signal recognition particle receptor; protein targeting	
CG11941	*skpC*	*skpC*	SCF complex; ubiquitin-dependent protein degradation	
CG12225	*Spt6*	*Spt6*	elongation of transcription; RNA maturation (exosome)	
CG32211	*TBP-associated factor 6*	*Taf6*	TFIID subunit; transcription	
CG12870	*Tia1-like*	*Tial*	RNA splicing	
CG11527	*Tiggrin*	*Tig*	integrin binding; signaling	zygotic mutations associated to melanotic masses and lamellocyte (tig^{A1}/tig^{X})
CG2762	*u-shaped*	*ush*	transcriptional co-repressor; transcription	zygotic mutation associated to lamellocytes [38] and melanotic masses (ush^{rev24}; ush^{rev24}/ush^{VX22})
CG17437	*will die slowly*	*wds*	MLL complex component; histone methylation; transcription	partner of a known melanotic tumor suppressor (*Ddb1* [47])
CG6197	*Xpa binding protein 2*	*Xab2*	RNA splicing	
