# Supplementary material for: An in vivo RNA interference screen identifies gene networks controlling Drosophila melanogaster blood cell homeostasis
Source: BMC Dev Biol. 2010 Jun 11;10:65. doi: 10.1186/1471-213X-10-65 (PMC2891661; doi:10.1186/1471-213X-10-65)
Supplement: Additional file 3 — Figure S3. Flip-out analysis of the expression pattern of sn-Gal and gcm-Gal4 in circulating larval blood cells. Blood cells smears from third instar larvae of the indicated genotypes were processed to reveal nuclear-GFP (in green) and P1/NimC1 (in red) expression by double fluorescent immuno-labeling. Nuclei were counterstained with DAPI (blue). [file 1471-213X-10-65-S3.PDF]

Additional Table S1

Results from the primary and secondary screens

CG_NO	*SYMBOL*	Insertions	Primary screen *srp-Gal4*	Primary screen *hmlΔ-Gal4*	Secondary screen (% tumors) *srp-Gal4*	Secondary screen (% tumors) *hmlΔ-Gal4*	Secondary screen (% tumors) *cg-Gal4*
CG10001	*AR-2*	2	neg.	neg.	nd	nd	nd
CG10006	CG10006	1	neg.	neg.	nd	nd	nd
CG10018	*Snm1*	1	neg.	neg.	nd	nd	nd
CG10033	*for*	2	pos.	pos.	0	0	16.5
CG10034	*tj*	2	neg.	neg.	nd	nd	nd
CG10041	CG10041	1	neg.	neg.	nd	nd	nd
CG10079	*Egfr*	2	neg.	neg.	nd	nd	nd
CG10086	CG10086	1	neg.	neg.	nd	nd	nd
CG10089	CG10089	1	neg.	neg.	nd	nd	nd
CG10093	*Cyp313a3*	1	pos.	neg.	1.3	0	0
CG10104	CG10104	1	neg.	neg.	nd	nd	nd
CG10117	*ttv*	2	neg.	pos.	1.7	0	15.6
CG10118	*ple*	1	neg.	neg.	nd	nd	nd
CG10134	*beat-Va*	2	pos.	neg.	0	0	0
CG10137	CG10137	1	pos.	neg.	0	0	0
CG10143	*Adgf-E*	1	neg.	neg.	nd	nd	nd
CG10149	*Rpn6*	1	neg.	neg.	nd	nd	nd
CG10174	*Ntf-2r*	2	neg.	neg.	nd	nd	nd
CG10176	CG10176	1	neg.	neg.	nd	nd	nd
CG10186	CG10186	1	neg.	neg.	nd	nd	nd
CG10194	CG10194	1	neg.	pos.	0	0	0
CG10198	*Nup98*	2	neg.	neg.	nd	nd	nd
CG10207	*NaPi-T*	2	neg.	neg.	nd	nd	nd
CG10223	*Top2*	1	neg.	neg.	nd	nd	nd
CG10264	CG10264	1	neg.	neg.	nd	nd	nd
CG10275	*kon*	1	neg.	neg.	nd	nd	nd
CG10278	*GATAe*	1	pos.	neg.	2.4	2.7	4.3
CG10295	*Pak*	1	pos.	neg.	24	0	leth
CG10336	CG10336	1	neg.	neg.	nd	nd	nd
CG10338	CG10338	2	neg.	neg.	nd	nd	nd
CG10352	CG10352	1	neg.	neg.	nd	nd	nd
CG10362	CG10362	2	pos.	neg.	6	0	3.7
CG10364	*msb1l*	2	neg.	neg.	nd	nd	nd
CG10366	CG10366	2	neg.	neg.	nd	nd	nd

CG10371	*Plip*	1	neg.	neg.	nd	nd	nd
CG10376	CG10376	2	neg.	pos.	0	0	0
CG10383	CG10383	1	neg.	neg.	nd	nd	nd
CG10385	*msl-1*	1	pos.	neg.	0	0	0
CG10424	CG10424	2	neg.	neg.	nd	nd	nd
CG10428	CG10428	2	neg.	pos.	0	0	0
CG10470	CG10470	2	neg.	neg.	nd	nd	nd
CG10472	CG10472	1	neg.	neg.	nd	nd	nd
CG10475	*Jon65Ai*	1	neg.	neg.	nd	nd	nd
CG10492	CG10492	1	pos.	neg.	1.4	1.8	0
CG10493	*Phlpp*	1	neg.	pos.	0	1.3	0
CG10522	*sti*	2	neg.	neg.	nd	nd	nd
CG10545	*Gbeta13F*	2	neg.	neg.	nd	nd	nd
CG1057	*MED31*	2	pos.	neg.	0	0	0
CG10572	*Cdk8*	1	neg.	neg.	nd	nd	nd
CG10579	*Eip63E*	1	neg.	neg.	nd	nd	nd
CG10584	CG10584	1	neg.	neg.	nd	nd	nd
CG10587	CG10587	2	neg.	neg.	nd	nd	nd
CG10588	CG10588	1	neg.	neg.	nd	nd	nd
CG10591	CG10591	1	neg.	neg.	nd	nd	nd
CG10593	*Acer*	1	neg.	neg.	0	0	0
CG10600	CG10600	1	neg.	neg.	nd	nd	nd
CG10602	CG10602	1	neg.	neg.	nd	nd	nd
CG10603	*mRpL13*	1	pos.	neg.	4	12	36
CG10616	CG10616	1	neg.	neg.	nd	nd	nd
CG10619	*tup*	2	neg.	neg.	nd	nd	nd
CG10637	*Nak*	1	neg.	neg.	nd	nd	nd
CG10655	*l(2)37Bb*	2	neg.	pos.	0	0	0
CG10658	*Hf*	2	neg.	neg.	nd	nd	nd
CG10663	CG10663	1	neg.	neg.	nd	nd	nd
CG10671	CG10671	2	neg.	neg.	nd	nd	nd
CG10695	*Pat1*	2	neg.	neg.	nd	nd	nd
CG10697	*Ddc*	2	pos.	pos.	1	0	0
CG10700	CG10700	1	pos.	neg.	0	0	1.7
CG10738	CG10738	2	neg.	neg.	nd	nd	nd
CG10746	*fok*	2	neg.	neg.	nd	nd	nd
CG10814	CG10814	2	neg.	neg.	nd	nd	nd
CG10823	*SIFR*	2	neg.	neg.	nd	nd	nd
CG10846	*dyn-p25*	1	neg.	pos.	2.8	5	0

CG10855	CG10855	1	neg.	neg.	nd	nd	nd
CG10863	CG10863	1	neg.	neg.	nd	nd	nd
CG10881	CG10881	1	neg.	neg.	nd	nd	nd
CG10898	CG10898	2	neg.	neg.	nd	nd	nd
CG10908	CG10908	2	pos.	pos.	0	0	0
CG10909	CG10909	2	pos.	pos.	1.2	1.6	0
CG10954	*Arc-p34*	2	pos.	pos.	0	2	4.8
CG10962	CG10962	2	neg.	neg.	nd	nd	nd
CG10984	CG10984	2	neg.	neg.	nd	nd	nd
CG1100	*Rpn5*	1	neg.	neg.	nd	nd	nd
CG1102	*MP1*	1	neg.	pos.	0	0	1.7
CG11035	CG11035	1	neg.	neg.	nd	nd	nd
CG11037	CG11037	1	neg.	neg.	nd	nd	nd
CG11044	CG11044	1	neg.	neg.	nd	nd	nd
CG11064	*Rfabg*	1	neg.	neg.	0	0	0
CG11076	CG11076	2	neg.	neg.	nd	nd	nd
CG11081	*plexA*	3	neg.	neg.	nd	nd	nd
CG11084	*pk*	1	neg.	pos.	0	0	0
CG11093	CG11093	1	neg.	neg.	nd	nd	nd
CG11095	CG11095	1	neg.	neg.	nd	nd	nd
CG11100	*Mes2*	2	pos.	neg.	0	0	0
CG11101	*pwn*	2	neg.	neg.	nd	nd	nd
CG11123	CG11123	1	neg.	neg.	nd	nd	nd
CG11124	*sPLA2*	2	neg.	neg.	nd	nd	nd
CG11125	CG11125	2	neg.	neg.	nd	nd	nd
CG11144	*mGluRA*	2	neg.	neg.	nd	nd	nd
CG11152	CG11152	2	neg.	neg.	nd	nd	nd
CG11165	CG11165	1	neg.	neg.	nd	nd	nd
CG11181	*cup*	1	neg.	pos.	0	0	0
CG11190	CG11190	1	pos.	neg.	1.4	0	3.9
CG11202	*org-1*	2	pos.	neg.	0	0	0
CG11210	CG11210	2	neg.	neg.	nd	nd	nd
CG11212	*Ptr*	1	neg.	neg.	nd	nd	nd
CG11295	*l(2)dtl*	1	neg.	pos.	4	11.3	23.6
CG11303	*TM4SF*	2	neg.	neg.	nd	nd	nd
CG11308	*sa*	2	pos.	neg.	0	0	0
CG11318	CG11318	2	neg.	neg.	nd	nd	nd
CG11326	*Tsp*	1	neg.	neg.	nd	nd	nd
CG11331	*Spn27A*	2	neg.	neg.	nd	nd	nd

CG11356	CG11356	2	neg.	pos.	0	0	0
CG11360	CG11360	1	neg.	neg.	nd	nd	nd
CG11364	*Cyp318a1*	1	neg.	neg.	nd	nd	nd
CG11371	*dbr*	1	neg.	neg.	nd	nd	nd
CG11387	*ct*	1	neg.	neg.	nd	nd	nd
CG11396	CG11396	2	neg.	neg.	nd	nd	nd
CG11405	*A3-3*	1	neg.	neg.	nd	nd	nd
CG11420	*png*	1	neg.	neg.	nd	nd	nd
CG11426	CG11426	2	pos.	neg.	0	1.4	0
CG11437	CG11437	2	neg.	neg.	nd	nd	nd
CG11440	*laza*	2	neg.	neg.	nd	nd	nd
CG11447	CG11447	1	neg.	neg.	nd	nd	nd
CG11448	CG11448	1	neg.	neg.	nd	nd	nd
CG11451	*Spc105R*	1	neg.	neg.	nd	nd	nd
CG11466	*Cyp9f2*	1	neg.	pos.	0	0	0
CG1147	*NPFR1*	2	neg.	neg.	nd	nd	nd
CG11495	*rasp*	2	neg.	neg.	nd	nd	nd
CG1152	*Gld*	2	neg.	neg.	nd	nd	nd
CG11522	*RpL6*	1	pos.	neg.	4.8	6.7	0
CG11527	*Tig*	1	pos.	neg.	0	0	12.1
CG11539	CG11539	2	neg.	neg.	nd	nd	nd
CG11576	CG11576	2	neg.	neg.	nd	nd	nd
CG11600	CG11600	2	neg.	neg.	0	0	0
CG11608	CG11608	2	neg.	pos.	0	0	0
CG11621	*Pi3K68D*	1	neg.	neg.	nd	nd	nd
CG11642	*TRAM*	2	neg.	neg.	nd	nd	nd
CG1165	*LysS*	1	neg.	neg.	nd	nd	nd
CG11664	CG11664	2	neg.	neg.	nd	nd	nd
CG11696	CG11696	2	pos.	neg.	0	1.4	0
CG11738	*l(1)G0004*	2	neg.	neg.	nd	nd	nd
CG11798	*chn*	2	neg.	neg.	nd	nd	nd
CG11802	CG11802	2	neg.	neg.	nd	nd	nd
CG11804	*ced-6*	1	neg.	neg.	nd	nd	nd
CG11816	CG11816	2	neg.	neg.	nd	nd	nd
CG11833	*Ssl2*	1	neg.	neg.	nd	nd	nd
CG11836	CG11836	1	neg.	neg.	nd	nd	nd
CG11837	CG11837	1	neg.	pos.	5	0	84.1
CG11841	CG11841	1	neg.	pos.	0	0	0
CG11842	CG11842	2	neg.	neg.	nd	nd	nd

CG11843	CG11843	2	neg.	neg.	nd	nd	nd
CG11856	*Nup358*	1	neg.	neg.	nd	nd	nd
CG11861	*cul-3*	1	neg.	neg.	nd	nd	nd
CG11865	CG11865	1	pos.	neg.	0	0	0
CG11866	CG11866	2	pos.	neg.	0	1.6	2
CG11870	CG11870	1	pos.	neg.	0	0	nd
CG11873	CG11873	1	neg.	neg.	nd	nd	nd
CG11882	CG11882	2	neg.	neg.	nd	nd	nd
CG11888	*Rpn2*	2	pos.	pos.	5	4	leth
CG11897	CG11897	1	neg.	neg.	nd	nd	nd
CG11898	CG11898	2	neg.	neg.	nd	nd	nd
CG11900	CG11900	1	pos.	neg.	0	0	0
CG11901	*Ef1gamma*	2	pos.	neg.	17.2	0	leth
CG11911	CG11911	2	neg.	neg.	0	0	0
CG11912	CG11912	1	pos.	pos.	2	3.3	0
CG11941	*skpC*	1	pos.	neg.	0	0	10.3
CG11942	*skpE*	1	neg.	neg.	nd	nd	nd
CG11981	*Prosbeta3*	1	neg.	neg.	nd	nd	nd
CG11990	*hyx*	1	pos.	neg.	55	8.3	7.7
CG1200	*Aplip1*	1	neg.	neg.	nd	nd	nd
CG12006	CG12006	1	neg.	neg.	nd	nd	nd
CG12007	CG12007	1	neg.	neg.	nd	nd	nd
CG12042	CG12042	1	neg.	neg.	nd	nd	nd
CG12057	CG12057	1	neg.	neg.	nd	nd	nd
CG12069	CG12069	2	neg.	neg.	nd	nd	nd
CG12072	*wts*	1	neg.	pos.	0	0	0
CG12079	CG12079	1	neg.	neg.	nd	nd	nd
CG12089	*beat-VI*	1	neg.	neg.	nd	nd	nd
CG12092	*NPC1b*	1	pos.	neg.	0	0	nd
CG12093	CG12093	2	neg.	neg.	nd	nd	nd
CG12116	CG12116	1	pos.	neg.	0	0	0
CG12119	CG12119	2	neg.	neg.	nd	nd	nd
CG12129	CG12129	1	neg.	neg.	nd	nd	nd
CG12139	CG42611	1	neg.	neg.	nd	nd	nd
CG12149	*c12.2*	2	pos.	neg.	2	0	0
CG12154	*oc*	2	pos.	pos.	67	9.4	39.4
CG12159	CG12159	2	pos.	neg.	0	0	0
CG12161	*Prosbeta2R2*	2	pos.	neg.	0	0	1.7
CG12169	*Ppm1*	1	neg.	neg.	nd	nd	nd

CG12172	*Spn43Aa*	2	neg.	neg.	nd	nd	nd
CG12177	CG12177	2	neg.	neg.	nd	nd	nd
CG12187	CG12187	1	neg.	neg.	nd	nd	nd
CG12201	CG12201	2	neg.	neg.	nd	nd	nd
CG12208	*Hr46*	1	neg.	neg.	nd	nd	nd
CG12217	*PpV*	1	pos.	neg.	0	0	0
CG12225	*Spt6*	1	pos.	neg.	32.1	1.4	41.4
CG12227	*skpF*	1	pos.	pos.	0	0	0
CG12229	CG12229	1	neg.	neg.	nd	nd	nd
CG12251	*AQP*	1	neg.	neg.	nd	nd	nd
CG12256	CG12256	2	neg.	neg.	nd	nd	nd
CG12276	*Aos1*	1	pos.	neg.	0	10	56.4
CG1228	*Ptpmeg*	2	neg.	neg.	nd	nd	nd
CG12290	CG12290	2	neg.	neg.	nd	nd	nd
CG12298	*sub*	1	neg.	neg.	nd	nd	nd
CG12301	CG12301	1	neg.	neg.	nd	nd	nd
CG12301	CG12301	2	neg.	neg.	nd	nd	nd
CG12306	*polo*	2	neg.	neg.	nd	nd	nd
CG12340	CG12340	1	pos.	neg.	0	0	0
CG12345	*Cha*	2	neg.	neg.	nd	nd	nd
CG12351	*deltaTry*	1	neg.	neg.	nd	nd	nd
CG12352	*san*	2	pos.	pos.	nd	nd	nd
CG12370	CG12370	2	neg.	neg.	nd	nd	nd
CG12375	CG12375	2	pos.	pos.	0	2.5	0
CG12384	CG12384	1	neg.	neg.	nd	nd	nd
CG1241	*Atg2*	1	neg.	neg.	nd	nd	nd
CG12505	*Arc1*	1	neg.	neg.	nd	nd	nd
CG12516	CG12516	2	neg.	neg.	nd	nd	nd
CG12519	CG12519	2	neg.	neg.	nd	nd	nd
CG12558	CG12558	1	neg.	neg.	nd	nd	nd
CG12581	CG12581	2	neg.	neg.	nd	nd	nd
CG12653	*btd*	2	pos.	pos.	37.3	0	leth
CG12661	CG12661	2	pos.	pos.	2.5	9	54.5
CG12664	*fend*	1	neg.	neg.	nd	nd	nd
CG12665	*Obp8a*	1	neg.	neg.	nd	nd	nd
CG12680	CG12680	2	neg.	pos.	0	0	0
CG12682	CG12682	2	neg.	neg.	nd	nd	nd
CG12715	CG12715	2	neg.	neg.	nd	nd	nd
CG12716	CG12716	1	neg.	neg.	nd	nd	nd

CG12717	CG12717	1	neg.	neg.	nd	nd	nd
CG12720	*Ten-a*	2	neg.	neg.	nd	nd	nd
CG12721	CG12721	2	neg.	neg.	nd	nd	nd
CG12723	CG12723	2	neg.	neg.	nd	nd	nd
CG12726	CG12726	1	neg.	neg.	nd	nd	nd
CG12734	CG12734	2	neg.	neg.	nd	nd	nd
CG12743	*otu*	1	neg.	neg.	nd	nd	nd
CG12765	CG12765	2	neg.	neg.	nd	nd	nd
CG12772	CG12772	2	pos.	neg.	14	0	75
CG12789	*santa-maria*	1	neg.	neg.	nd	nd	nd
CG12822	CG12822	2	pos.	neg.	0	0	2
CG12825	CG12825	1	neg.	neg.	nd	nd	nd
CG12831	CG12831	1	neg.	neg.	nd	nd	nd
CG12832	*Tsp42Eq*	2	pos.	neg.	0	0	0
CG12833	*esn*	2	neg.	neg.	nd	nd	nd
CG12838	*Tsp42Eo*	2	neg.	neg.	nd	nd	nd
CG12839	*Tsp42En*	2	pos.	pos.	0	0	0
CG12841	*Tsp42Ek*	2	neg.	pos.	0	0.7	0
CG12847	*Tsp42Ec*	1	neg.	neg.	nd	nd	nd
CG12857	CG12857	1	neg.	neg.	nd	nd	nd
CG12866	CG12866	2	neg.	neg.	nd	nd	nd
CG12870	CG34362	2	pos.	neg.	30	0	65.2
CG12885	CG12885	2	pos.	pos.	0	0	0
CG12908	*Ndg*	2	neg.	neg.	nd	nd	nd
CG12909	CG12909	2	pos.	neg.	0	0	0
CG12910	CG12910	1	neg.	neg.	nd	nd	nd
CG1295	CG1295	2	neg.	neg.	nd	nd	nd
CG12951	CG12951	2	neg.	neg.	nd	nd	nd
CG12954	*mRpL41*	2	neg.	pos.	0	0	83.6
CG12960	*Ir52a*	2	neg.	neg.	nd	nd	nd
CG12964	CG12964	1	neg.	neg.	nd	nd	nd
CG12975	CG12975	1	neg.	neg.	nd	nd	nd
CG12983	CG12983	2	neg.	neg.	nd	nd	nd
CG12984	CG12984	2	neg.	neg.	nd	nd	nd
CG1299	CG1299	1	neg.	neg.	nd	nd	nd
CG13077	CG13077	2	neg.	neg.	nd	nd	nd
CG13155	CG13155	1	pos.	neg.	0	0	0
CG13160	CG13160	1	neg.	neg.	nd	nd	nd
CG13189	CG13189	1	neg.	pos.	0	3	0

CG13194	*pyr*	1	pos.	neg.	28	0	1.8
CG13243	CG13243	1	neg.	neg.	nd	nd	nd
CG13247	CG13247	1	pos.	neg.	0	0	1.7
CG13251	CG13251	1	neg.	neg.	nd	nd	nd
CG13271	*Ugt36Bb*	1	neg.	neg.	nd	nd	nd
CG13272	CG13272	1	neg.	neg.	nd	nd	nd
CG13280	CG13280	2	neg.	neg.	nd	nd	nd
CG13284	CG13284	1	pos.	neg.	0	0	0
CG13325	CG13325	1	neg.	neg.	nd	nd	nd
CG13335	CG13335	2	neg.	pos.	0	3	0
CG13337	CG13337	2	neg.	neg.	nd	nd	nd
CG13338	*Cpr50Ca*	1	neg.	neg.	nd	nd	nd
CG13339	CG13339	2	neg.	neg.	nd	nd	nd
CG13345	*tum*	1	neg.	neg.	nd	nd	nd
CG13351	*RN-tre*	1	pos.	neg.	0	0	1.5
CG13379	*Sgf11*	1	neg.	neg.	nd	nd	nd
CG1342	CG1342	1	neg.	neg.	nd	nd	nd
CG1343	*Sp1*	2	neg.	neg.	nd	nd	nd
CG13549	*yip3*	1	neg.	neg.	nd	nd	nd
CG13566	CG13566	2	pos.	neg.	0	0	0
CG13579	CG13579	2	neg.	neg.	0	0	0
CG1358	CG1358	2	pos.	neg.	0	0	0
CG13641	CG13641	2	neg.	neg.	nd	nd	nd
CG13651	*danr*	2	neg.	neg.	nd	nd	nd
CG13698	CG13698	2	neg.	neg.	nd	nd	nd
CG13733	CG13733	2	neg.	neg.	nd	nd	nd
CG13758	*pdfr*	2	neg.	neg.	nd	nd	nd
CG13766	CG13766	1	neg.	neg.	nd	nd	nd
CG13894	CG13894	1	neg.	neg.	nd	nd	nd
CG13942	*Shroom*	1	neg.	neg.	nd	nd	nd
CG1395	*stg*	1	neg.	neg.	nd	nd	nd
CG13977	*Cyp6a18*	2	neg.	neg.	nd	nd	nd
CG13978	CG13978	1	neg.	neg.	nd	nd	nd
CG1399	CG1399	2	neg.	neg.	nd	nd	nd
CG13995	CG13995	2	neg.	neg.	nd	nd	nd
CG1401	*cul-5*	2	neg.	neg.	0	0	0
CG14026	*tkv*	2	neg.	neg.	nd	nd	nd
CG14029	*vri*	1	neg.	neg.	nd	nd	nd
CG1406	*U2A*	1	neg.	neg.	nd	nd	nd

CG14077	CG14077	1	neg.	neg.	nd	nd	nd
CG14082	CG14082	2	neg.	pos.	2	0	0
CG14085	CG14085	1	neg.	neg.	nd	nd	nd
CG14086	CG14086	2	neg.	neg.	nd	nd	nd
CG14087	CG14087	1	neg.	neg.	nd	nd	nd
CG1409	CG1409	1	neg.	neg.	nd	nd	nd
CG14100	CG14100	2	neg.	pos.	8	2.4	3.6
CG14102	CG14102	1	neg.	pos.	0	4.9	0
CG14187	CG14187	1	neg.	neg.	nd	nd	nd
CG14207	CG14207	2	neg.	neg.	nd	nd	nd
CG14211	*MKP-4*	2	neg.	neg.	nd	nd	nd
CG14222	CG14222	1	neg.	neg.	nd	nd	nd
CG14230	CG14230	2	pos.	neg.	5	2.9	70.7
CG14291	CG14291	2	neg.	neg.	nd	nd	nd
CG1433	*Atu*	1	neg.	neg.	nd	nd	nd
CG14355	CG14355	1	neg.	neg.	nd	nd	nd
CG14375	CG14375	1	neg.	neg.	nd	nd	nd
CG1438	*Cyp4c3*	1	neg.	pos.	0	4.1	0.9
CG14381	*timeout*	1	neg.	neg.	0	0	0
CG14384	CG14384	2	neg.	neg.	nd	nd	nd
CG14387	*d-cup*	1	neg.	neg.	nd	nd	nd
CG14390	*beat-Vc*	2	neg.	neg.	nd	nd	nd
CG1440	CG1440	2	neg.	neg.	nd	nd	nd
CG14401	CG14401	1	neg.	neg.	nd	nd	nd
CG14427	CG14427	2	neg.	neg.	nd	nd	nd
CG1443	CG1443	1	neg.	neg.	nd	nd	nd
CG14437	*COQ7*	2	pos.	neg.	0	0	0
CG14439	CG14439	2	neg.	neg.	nd	nd	nd
CG14441	CG14441	1	neg.	neg.	nd	nd	nd
CG14446	CG14446	1	pos.	neg.	3.2	0	3.2
CG14509	CG14509	1	neg.	neg.	nd	nd	nd
CG14512	CG14512	1	pos.	neg.	5.6	0	13.2
CG14513	*yemalpha*	1	neg.	neg.	nd	nd	nd
CG14516	CG14516	1	neg.	neg.	nd	nd	nd
CG14527	CG14527	1	neg.	neg.	nd	nd	nd
CG1453	*Klp10A*	1	neg.	neg.	nd	nd	nd
CG14574	CG14574	1	neg.	neg.	nd	nd	nd
CG1458	CG1458	1	neg.	neg.	nd	nd	nd
CG14619	CG14619	1	neg.	neg.	nd	nd	nd

CG14625	CG14625	2	neg.	neg.	nd	nd	nd
CG14626	CG14626	1	neg.	neg.	nd	nd	nd
CG14637	*abs*	1	neg.	neg.	nd	nd	nd
CG14642	CG14642	1	neg.	neg.	nd	nd	nd
CG1469	*Fer2LCH*	1	neg.	neg.	nd	nd	nd
CG1471	*CDase*	1	neg.	neg.	nd	nd	nd
CG14750	*Vps25*	2	neg.	pos.	1.8	0	0
CG14760	CG14760	2	neg.	neg.	nd	nd	nd
CG14762	CG14762	1	pos.	neg.	0	0	0
CG14763	CG14763	1	neg.	pos.	0	0	0
CG1488	*Cyp311a1*	1	neg.	neg.	nd	nd	nd
CG14884	*CSN5*	2	neg.	pos.	0	0	0
CG14895	*Pak3*	1	neg.	neg.	nd	nd	nd
CG1492	CG1492	2	neg.	neg.	nd	nd	nd
CG1495	*CaMKI*	1	neg.	neg.	nd	nd	nd
CG1499	CG1499	1	neg.	neg.	nd	nd	nd
CG1499	CG1499	1	neg.	neg.	nd	nd	nd
CG14990	CG14990	1	neg.	neg.	nd	nd	nd
CG14992	*Ack*	2	neg.	neg.	nd	nd	nd
CG14999	*RfC4*	2	pos.	pos.	25.9	0	23
CG1505	*gd*	1	neg.	pos.	0	0	0.8
CG15072	CG15072	2	neg.	neg.	nd	nd	nd
CG1511	*Eph*	2	neg.	neg.	nd	nd	nd
CG15118	CG15118	1	neg.	neg.	nd	nd	nd
CG1512	*cul-2*	1	neg.	neg.	nd	nd	nd
CG15136	CG15136	1	neg.	neg.	nd	nd	nd
CG15155	CG15155	2	neg.	pos.	0	3.6	0
CG15160	CG15160	1	neg.	neg.	nd	nd	nd
CG15161	CG15161	2	neg.	neg.	nd	nd	nd
CG15173	CG15173	2	neg.	neg.	nd	nd	nd
CG1520	*WASp*	1	neg.	neg.	nd	nd	nd
CG15247	CG15247	1	pos.	neg.	1.4	3.6	13.3
CG15252	CG15252	1	neg.	neg.	nd	nd	nd
CG15253	CG15253	2	neg.	neg.	nd	nd	nd
CG15255	CG15255	1	neg.	neg.	nd	nd	nd
CG15262	CG15262	1	neg.	neg.	nd	nd	nd
CG1527	*RpS14b*	2	neg.	neg.	nd	nd	nd
CG15280	*CR15280*	2	neg.	neg.	nd	nd	nd
CG15305	*flw*	1	neg.	neg.	nd	nd	nd

CG15314	CG15314	1	pos.	pos.	12.3	1.9	leth
CG15319	*nej*	1	neg.	neg.	nd	nd	nd
CG1532	CG1532	2	neg.	neg.	nd	nd	nd
CG15343	CG15343	2	neg.	neg.	nd	nd	nd
CG15347	CG15347	2	pos.	neg.	0	0	11.1
CG15350	*Cp7Fb*	2	neg.	neg.	nd	nd	nd
CG15368	CG15368	1	neg.	neg.	nd	nd	nd
CG15369	CG15369	1	pos.	neg.	0	0	0
CG15370	CG15370	1	neg.	neg.	nd	nd	nd
CG15412	CG15412	1	neg.	neg.	nd	nd	nd
CG15436	CG15436	1	pos.	neg.	0	0	0
CG15437	*morgue*	2	neg.	neg.	nd	nd	nd
CG15444	*ine*	1	neg.	neg.	nd	nd	nd
CG15470	CG42594	2	neg.	neg.	nd	nd	nd
CG15504	*dmrt99B*	1	neg.	neg.	nd	nd	nd
CG1569	*rod*	1	neg.	neg.	nd	nd	nd
CG15697	*RpS30*	1	pos.	pos.	2.9	0	0
CG15698	*Oamb*	2	neg.	neg.	nd	nd	nd
CG1571	CG1571	1	neg.	neg.	nd	nd	nd
CG15717	CG15717	1	neg.	neg.	nd	nd	nd
CG15717	CG15717	2	neg.	neg.	nd	nd	nd
CG15721	CG15721	1	neg.	neg.	nd	nd	nd
CG15727	CG15727	1	pos.	pos.	1.5	0	1
CG15728	CG15728	2	neg.	neg.	nd	nd	nd
CG15730	CG15730	2	neg.	neg.	nd	nd	nd
CG15738	CG15738	2	neg.	pos.	0	0	1.8
CG15738	CG15738	2	neg.	neg.	nd	nd	nd
CG15739	CG15739	1	neg.	neg.	nd	nd	nd
CG15743	CG15743	1	neg.	neg.	nd	nd	nd
CG15748	CG15748	2	neg.	neg.	nd	nd	nd
CG15749	*dmrt11E*	1	neg.	neg.	nd	nd	nd
CG15754	CG15754	2	neg.	neg.	nd	nd	nd
CG15764	CG15764	1	neg.	neg.	nd	nd	nd
CG15766	CG15766	2	neg.	neg.	nd	nd	nd
CG15768	CG42264	2	neg.	neg.	nd	nd	nd
CG15768	CG42264	2	pos.	neg.	0	0	0
CG15769	CG42264	1	neg.	neg.	nd	nd	nd
CG1577	*mRpL52*	1	pos.	pos.	1	5.2	86.8
CG15771	CG15771	1	neg.	neg.	nd	nd	nd

CG15773	CG15773	1	neg.	neg.	nd	nd	nd
CG15784	CG15784	1	pos.	pos.	7.5	6	0
CG1582	CG1582	2	neg.	neg.	nd	nd	nd
CG15927	CG15927	1	pos.	neg.	0	0	1.5
CG15929	*lin-52*	1	pos.	pos.	0	2.4	3.3
CG1602	CG1602	1	neg.	neg.	nd	nd	nd
CG1603	CG1603	1	neg.	neg.	nd	nd	nd
CG1607	CG1607	2	neg.	neg.	nd	nd	nd
CG1609	*Gcn2*	2	neg.	neg.	nd	nd	nd
CG1616	*dpa*	2	neg.	neg.	nd	nd	nd
CG1624	*dpld*	2	neg.	neg.	nd	nd	nd
CG1629	*yellow-h*	1	pos.	neg.	0	0	0
CG1662	CG1662	1	neg.	neg.	nd	nd	nd
CG16705	*SPE*	2	neg.	neg.	nd	nd	nd
CG16718	CG16718	2	pos.	neg.	0	0	nd
CG16724	*tra*	1	neg.	neg.	nd	nd	nd
CG1673	CG1673	1	neg.	neg.	nd	nd	nd
CG1674	CG1674	2	neg.	neg.	nd	nd	nd
CG16747	*Oda*	1	neg.	neg.	nd	nd	nd
CG16766	*TyrRII*	1	neg.	neg.	nd	nd	nd
CG16788	*RnpS1*	1	neg.	neg.	nd	nd	nd
CG16796	*Hmgs*	2	neg.	neg.	0	0	0
CG16799	CG16799	2	pos.	neg.	0	0	0
CG16820	CG16820	1	pos.	pos.	1.3	1	1.8
CG16827	*alphaPS4*	1	neg.	neg.	nd	nd	nd
CG16904	CG16904	2	neg.	neg.	nd	nd	nd
CG16908	CG16908	2	neg.	neg.	nd	nd	nd
CG16935	CG16935	1	neg.	neg.	nd	nd	nd
CG16973	*msn*	2	pos.	neg.	4	0	31.6
CG16983	*skpA*	2	neg.	neg.	nd	nd	nd
CG1699	*Tsp66A*	2	neg.	neg.	nd	nd	nd
CG16993	*in*	2	neg.	neg.	nd	nd	nd
CG16996	CG16996	1	neg.	neg.	nd	nd	nd
CG16997	CG16997	2	neg.	neg.	nd	nd	nd
CG16998	CG16998	3	neg.	pos.	0	1.6	0
CG17012	CG17012	2	neg.	neg.	nd	nd	nd
CG17027	CG17027	2	neg.	neg.	nd	nd	nd
CG17029	CG17029	2	neg.	neg.	nd	nd	nd
CG17064	*mars*	1	neg.	neg.	nd	nd	nd

CG17084	*mthl9*	1	neg.	neg.	nd	nd	nd
CG17100	*cwo*	1	neg.	neg.	nd	nd	nd
CG17119	CG17119	1	neg.	neg.	nd	nd	nd
CG17122	CG17122	1	neg.	neg.	nd	nd	nd
CG17131	*SP71*	1	pos.	neg.	0	0	0
CG17161	*grp*	1	neg.	pos.	0	0	0
CG17172	*ATbp*	2	neg.	neg.	nd	nd	nd
CG17176	*ACXA*	1	neg.	neg.	nd	nd	nd
CG17218	CG17218	1	neg.	neg.	nd	nd	nd
CG17245	*plexB*	1	neg.	neg.	nd	nd	nd
CG17293	CG17293	1	pos.	neg.	4.9	0	0.9
CG17327	CG17327	1	pos.	neg.	0	0	0
CG17328	CG17328	1	neg.	neg.	nd	nd	nd
CG17331	CG17331	1	pos.	neg.	40	4	leth
CG17342	*Lk6*	1	neg.	neg.	nd	nd	nd
CG17348	*drl*	2	neg.	neg.	nd	nd	nd
CG17352	CG17352	1	neg.	neg.	nd	nd	nd
CG1736	*Prosalpha3T*	2	neg.	neg.	nd	nd	nd
CG17437	*wds*	1	pos.	neg.	0	0	52.4
CG17453	*Cyp317a1*	1	neg.	neg.	nd	nd	nd
CG17477	CG17477	1	neg.	neg.	nd	nd	nd
CG17487	*tankyrase*	1	neg.	neg.	nd	nd	nd
CG17489	*RpL5*	2	pos.	pos.	nd	nd	nd
CG17498	*mad2*	1	neg.	neg.	nd	nd	nd
CG1750	CG1750	1	neg.	neg.	nd	nd	nd
CG17514	CG17514	1	neg.	neg.	nd	nd	nd
CG17520	*CkIIalpha*	1	neg.	neg.	nd	nd	nd
CG17540	*Spf45*	1	neg.	neg.	nd	nd	nd
CG17559	*dnt*	1	neg.	neg.	nd	nd	nd
CG17568	CG17568	1	neg.	neg.	nd	nd	nd
CG17572	CG17572	2	neg.	neg.	nd	nd	nd
CG17596	*S6kII*	1	neg.	neg.	nd	nd	nd
CG17598	CG17598	1	neg.	neg.	nd	nd	nd
CG17610	*grk*	2	neg.	neg.	nd	nd	nd
CG17611	*eIF6*	2	neg.	neg.	nd	nd	nd
CG17617	*RhoGAP1A*	1	neg.	pos.	0	1.8	1.1
CG1762	*betaInt-nu*	1	neg.	neg.	nd	nd	nd
CG1763	*nod*	1	neg.	pos.	0	0	0
CG17646	CG17646	1	pos.	neg.	0	0	53.2

CG1765	*EcR*	2	pos.	neg.	3.7	0	0
CG17707	CG17707	1	neg.	neg.	nd	nd	nd
CG1771	*mew*	1	neg.	neg.	nd	nd	nd
CG17711	CG31937	2	neg.	neg.	nd	nd	nd
CG17712	CG17712	2	neg.	neg.	nd	nd	nd
CG17717	CG17717	1	neg.	neg.	nd	nd	nd
CG17736	*schuy*	1	neg.	neg.	nd	nd	nd
CG17758	CG42265	2	neg.	neg.	nd	nd	nd
CG17760	CG17760	2	neg.	neg.	nd	nd	nd
CG17764	CG17764	1	neg.	pos.	0	0	1.4
CG1780	*Idgf4*	2	neg.	neg.	nd	nd	nd
CG17806	CG17806	2	neg.	pos.	0	0	0
CG1787	*Hexo2*	1	neg.	neg.	nd	nd	nd
CG17883	CG17883	2	neg.	neg.	nd	nd	nd
CG17904	CG17904	1	neg.	neg.	nd	nd	nd
CG1792	CG1792	1	neg.	neg.	nd	nd	nd
CG17923	*JYalpha*	1	neg.	neg.	nd	nd	nd
CG17960	*RhoGAP1A*	1	neg.	neg.	nd	nd	nd
CG17975	*sut2*	1	neg.	pos.	0	0	2.9
CG17976	*sut3*	2	neg.	neg.	nd	nd	nd
CG18039	*KaiRIA*	2	pos.	neg.	2	8	6.8
CG1804	*kek6*	2	pos.	pos.	0	0	1.9
CG18063	CG18063	2	neg.	neg.	nd	nd	nd
CG18104	*arg*	2	neg.	neg.	nd	nd	nd
CG18130	CG18130	1	neg.	neg.	nd	nd	nd
CG18155	CG18155	1	neg.	neg.	nd	nd	nd
CG18174	*Rpn11*	2	neg.	neg.	nd	nd	nd
CG18178	CG18178	1	neg.	neg.	nd	nd	nd
CG18180	CG18180	1	neg.	neg.	nd	nd	nd
CG1819	CG34120	2	neg.	pos.	0	4.1	0
CG1821	*RpL31*	2	neg.	neg.	nd	nd	nd
CG18223	CG18223	1	neg.	neg.	nd	nd	nd
CG18265	CG18265	2	neg.	neg.	nd	nd	nd
CG18279	*IM10*	1	neg.	neg.	nd	nd	nd
CG18293	CG15333	2	neg.	neg.	nd	nd	nd
CG18294	CG18294	3	neg.	pos.	0	0	1.5
CG1832	CG1832	1	neg.	pos.	21	7.7	9.3
CG18330	*Cct2*	1	neg.	neg.	0	0	0
CG18341	*Prosbeta2R1*	1	neg.	neg.	nd	nd	nd

CG18372	*AttB*	1	neg.	neg.	nd	nd	nd
CG18396	*Mst98Cb*	2	neg.	neg.	nd	nd	nd
CG18402	*InR*	1	neg.	neg.	nd	nd	nd
CG18412	*ph-p*	1	neg.	neg.	nd	nd	nd
CG18472	CG18472	2	neg.	neg.	nd	nd	nd
CG18478	CG18478	2	pos.	pos.	0	0	0
CG18495	*Prosalpha1*	2	pos.	pos.	26.7	4.8	leth
CG18507	CG18507	2	neg.	pos.	0	0	0
CG18557	CG18557	2	neg.	neg.	nd	nd	nd
CG18563	CG18563	2	neg.	neg.	nd	nd	nd
CG1857	*nec*	1	pos.	pos.	21.5	17.5	leth
CG1859	*Spn43Ad*	1	neg.	neg.	nd	nd	nd
CG18639	*NPFR76F*	2	neg.	neg.	nd	nd	nd
CG18657	*NetA*	2	neg.	neg.	nd	nd	nd
CG1873	*Ef1alpha100E*	1	neg.	pos.	9.5	2.5	18.4
CG18741	*DopR2*	1	neg.	neg.	nd	nd	nd
CG1877	*lin19*	1	neg.	neg.	0	0	0
CG18812	CG18812	2	neg.	neg.	nd	nd	nd
CG18815	CG18815	2	neg.	neg.	nd	nd	nd
CG18858	CG18858	1	neg.	neg.	nd	nd	nd
CG1891	*sax*	1	neg.	neg.	nd	nd	nd
CG1893	*scramb2*	2	pos.	neg.	0	0	4
CG1896	CG1896	1	neg.	neg.	nd	nd	nd
CG1901	*mav*	1	neg.	neg.	nd	nd	nd
CG1906	*alph*	1	neg.	neg.	nd	nd	nd
CG1924	CG1924	1	pos.	neg.	8	0	3.2
CG1941	CG1941	1	neg.	neg.	nd	nd	nd
CG1950	CG1950	1	neg.	neg.	nd	nd	nd
CG1951	CG1951	2	neg.	neg.	nd	nd	nd
CG1954	*Pkc98E*	2	neg.	neg.	nd	nd	nd
CG1958	CG1958	1	neg.	neg.	nd	nd	nd
CG1973	*yata*	2	neg.	neg.	nd	nd	nd
CG1994	*l(1)G0020*	2	pos.	neg.	12	0	17.5
CG1998	CG1998	1	neg.	neg.	nd	nd	nd
CG2010	CG2010	1	pos.	neg.	5.6	0	5.9
CG2045	*Ser7*	1	neg.	neg.	nd	nd	nd
CG2048	*dco*	1	pos.	neg.	8.9	0	51.4
CG2052	CG2052	1	pos.	neg.	1.6	0	0
CG2054	*Cht2*	1	pos.	neg.	0	0	0

CG2056	*spirit*	2	neg.	neg.	nd	nd	nd
CG2071	*Ser6*	2	neg.	neg.	nd	nd	nd
CG2096	*flw*	1	pos.	pos.	0	0	1.6
CG2105	*Corin*	1	neg.	neg.	nd	nd	nd
CG2114	*FR*	2	neg.	neg.	nd	nd	nd
CG2125	*ci*	2	neg.	neg.	nd	nd	nd
CG2126	CG2126	2	neg.	neg.	nd	nd	nd
CG2137	CG2137	1	neg.	neg.	nd	nd	nd
CG2160	*Socs44A*	1	neg.	pos.	0	0	1.9
CG2168	*RpS3A*	2	neg.	neg.	nd	nd	nd
CG2187	CG2187	2	neg.	neg.	nd	nd	nd
CG2190	*hep*	2	pos.	pos.	0	0	0
CG2191	*Smvt*	2	neg.	neg.	nd	nd	nd
CG2199	CG2199	2	neg.	pos.	0	0	0
CG2200	CG2200	1	neg.	neg.	nd	nd	nd
CG2210	*awd*	1	neg.	neg.	nd	nd	nd
CG2217	CG2217	2	neg.	neg.	nd	nd	nd
CG2218	CG2218	2	pos.	neg.	0	0	0
CG2229	*Jon99Fii*	2	neg.	neg.	nd	nd	nd
CG2241	*Rpt6R*	1	neg.	neg.	nd	nd	nd
CG2275	*Jra*	1	neg.	neg.	nd	nd	nd
CG2286	*ND75*	2	pos.	pos.	8	5.2	92.9
CG2310	CG2310	2	neg.	pos.	0	0	0
CG2316	CG2316	2	pos.	neg.	0	0	0
CG2374	*lbm*	1	pos.	neg.	0	0	0
CG2411	*ptc*	2	neg.	pos.	1.8	0	4.3
CG2453	CG2453	1	neg.	neg.	nd	nd	nd
CG2522	*Gtp-bp*	1	pos.	pos.	18	21.4	leth
CG2560	*Cpr11A*	1	neg.	neg.	nd	nd	nd
CG2574	CG2574	2	neg.	neg.	nd	nd	nd
CG2621	*sgg*	2	neg.	neg.	nd	nd	nd
CG2655	*HLH3B*	2	neg.	neg.	nd	nd	nd
CG2682	*d4*	1	pos.	pos.	0	0	0
CG2750	CG2750	1	neg.	neg.	nd	nd	nd
CG2762	*ush*	2	pos.	pos.	40	17	67.7
CG2767	CG2767	2	neg.	neg.	nd	nd	nd
CG2788	*Dot*	2	neg.	neg.	nd	nd	nd
CG2835	*G-salpha60A*	1	neg.	neg.	nd	nd	nd
CG2845	*phl*	2	neg.	neg.	nd	nd	nd

CG2848	*Trn-SR*	1	neg.	neg.	nd	nd	nd
CG2906	CG2906	2	neg.	neg.	nd	nd	nd
CG2909	CG2909	2	neg.	neg.	nd	nd	nd
CG2916	*Sep5*	1	neg.	neg.	0	0	0
CG2943	CG2943	2	pos.	neg.	0	0	3.8
CG2948	*rev7*	2	neg.	neg.	nd	nd	nd
CG2969	*Atet*	1	neg.	pos.	nd	nd	nd
CG2974	CG2974	1	pos.	neg.	0	0	0
CG2976	CG2976	2	neg.	neg.	nd	nd	nd
CG2984	*Pp2C1*	2	neg.	neg.	nd	nd	nd
CG2985	*Yp1*	1	neg.	neg.	nd	nd	nd
CG2993	CG2993	1	neg.	neg.	nd	nd	nd
CG2993	CG2993	2	neg.	pos.	0	0	0
CG30022	CG30022	2	neg.	neg.	nd	nd	nd
CG3003	CG3003	1	neg.	neg.	nd	nd	nd
CG3004	CG3004	2	neg.	neg.	nd	nd	nd
CG30043	CG30043	2	pos.	neg.	0	0	0
CG30045	*Cpr49Aa*	2	neg.	neg.	nd	nd	nd
CG30049	CG30049	1	neg.	neg.	nd	nd	nd
CG30051	CG30051	1	neg.	neg.	nd	nd	nd
CG30054	CG30054	2	neg.	neg.	nd	nd	nd
CG30060	CG30060	1	neg.	neg.	nd	nd	nd
CG30062	CG30062	2	neg.	neg.	nd	nd	nd
CG30072	*Obp50c*	1	pos.	neg.	0	0	0
CG30075	CG30075	2	neg.	neg.	nd	nd	nd
CG30076	CG30076	2	neg.	neg.	nd	nd	nd
CG30077	CG30077	1	neg.	neg.	nd	nd	nd
CG30091	CG30091	2	neg.	neg.	nd	nd	nd
CG3011	CG3011	1	neg.	neg.	nd	nd	nd
CG30148	CG30148	2	neg.	neg.	nd	nd	nd
CG30152	CG30152	2	pos.	neg.	0	4	0
CG30156	CG30156	2	neg.	neg.	nd	nd	nd
CG30156	CG30156	1	pos.	neg.	3.5	0	57.7
CG30160	CG30160	2	neg.	neg.	nd	nd	nd
CG30194	CG30194	1	neg.	neg.	nd	nd	nd
CG3021	CG3021	2	neg.	neg.	nd	nd	nd
CG30222	CG30222	2	neg.	neg.	nd	nd	nd
CG3024	*torp4a*	2	neg.	neg.	nd	nd	nd
CG30334	CG30334	2	pos.	neg.	0	0	0

CG30371	CG30371	2	neg.	neg.	nd	nd	nd
CG30379	CG30379	1	neg.	neg.	nd	nd	nd
CG30382	CG30382	2	pos.	pos.	0	0	0
CG30383	CG30383	1	neg.	neg.	nd	nd	nd
CG30426	*egg*	1	neg.	neg.	nd	nd	nd
CG30464	*Ir52d*	1	neg.	neg.	nd	nd	nd
CG30468	*Ir52c*	1	neg.	neg.	nd	nd	nd
CG30475	CG30475	1	neg.	neg.	nd	nd	nd
CG3048	*Traf4*	2	neg.	neg.	nd	nd	nd
CG30480	CG30480	2	pos.	neg.	0	0	0
CG30496	CG30496	1	neg.	neg.	nd	nd	nd
CG3050	*Cyp6d5*	1	neg.	neg.	nd	nd	nd
CG30502	CG30502	2	neg.	neg.	nd	nd	nd
CG3051	*SNF1A*	2	neg.	pos.	0	0	0
CG3057	*colt*	1	neg.	neg.	nd	nd	nd
CG3066	*Sp7*	1	pos.	neg.	0	0	nd
CG3074	CG3074	1	neg.	neg.	nd	nd	nd
CG3077	CG3077	1	neg.	neg.	nd	nd	nd
CG31004	CG31004	1	neg.	pos.	0	0	0
CG31044	CG31044	2	pos.	neg.	13.6	0	1.8
CG31053	CG31053	1	neg.	neg.	nd	nd	nd
CG31055	CG31055	1	neg.	neg.	nd	nd	nd
CG31062	*side*	1	neg.	neg.	nd	nd	nd
CG31063	CG31063	1	neg.	pos.	0	1.5	0
CG31092	*LpR2*	1	pos.	pos.	3	0	0
CG31094	*LpR1*	1	neg.	neg.	nd	nd	nd
CG31150	CG31150	1	neg.	neg.	nd	nd	nd
CG3117	CG3117	1	neg.	neg.	nd	nd	nd
CG31256	*Brf*	1	pos.	pos.	15.6	3.6	50.7
CG31298	*beat-Vb*	1	neg.	neg.	nd	nd	nd
CG31347	CG31347	1	pos.	neg.	2	0	0
CG31359	*Hsp70Bb*	1	pos.	neg.	7.1	7.6	leth
CG31367	CG31367	2	neg.	neg.	nd	nd	nd
CG31388	CG31388	1	neg.	neg.	nd	nd	nd
CG31421	*Tak1l*	1	neg.	neg.	nd	nd	nd
CG31426	*ligatin*	1	neg.	neg.	nd	nd	nd
CG31449	*Hsp70Ba*	1	neg.	neg.	nd	nd	nd
CG31469	CG31469	1	neg.	neg.	nd	nd	nd
CG31633	CG31633	1	neg.	neg.	nd	nd	nd

CG3164	CG3164	1	neg.	neg.	nd	nd	nd
CG31659	CG31659	1	neg.	neg.	nd	nd	nd
CG3166	*aop*	1	neg.	neg.	nd	nd	nd
CG31689	CG31689	1	neg.	neg.	nd	nd	nd
CG31694	CG31694	1	pos.	neg.	2.6	0	1.2
CG3171	*Tre1*	2	neg.	neg.	nd	nd	nd
CG31714	CG31714	1	neg.	neg.	nd	nd	nd
CG31731	CG31731	1	neg.	pos.	0	0	nd
CG31748	*Gr36c*	2	neg.	neg.	nd	nd	nd
CG31782	CG31782	1	neg.	neg.	nd	nd	nd
CG31800	CG31800	2	neg.	neg.	nd	nd	nd
CG31803	CG31803	1	neg.	neg.	nd	nd	nd
CG31805	CG31805	1	neg.	neg.	nd	nd	nd
CG31806	CG31806	1	neg.	pos.	0	0	4
CG31810	CG31810	1	neg.	neg.	nd	nd	nd
CG31821	CG31821	2	pos.	neg.	0	0	0
CG31823	CG31823	2	neg.	neg.	nd	nd	nd
CG31827	CG31827	1	neg.	neg.	nd	nd	nd
CG31829	*cul-3*	1	neg.	neg.	nd	nd	nd
CG31836	CG31836	1	neg.	neg.	nd	nd	nd
CG31918	CG31918	1	neg.	neg.	nd	nd	nd
CG31954	CG31954	1	neg.	neg.	nd	nd	nd
CG31999	CG31999	2	neg.	neg.	nd	nd	nd
CG3200	*Reg-2*	2	neg.	neg.	nd	nd	nd
CG32005	*pan*	2	neg.	neg.	nd	nd	nd
CG32009	*CR32009*	1	neg.	neg.	nd	nd	nd
CG32010	*CR32010*	1	neg.	neg.	nd	nd	nd
CG32011	*CR32011*	1	pos.	pos.	0	0	1.5
CG32018	*Zyx102EF*	1	neg.	neg.	nd	nd	nd
CG32019	*bt*	2	neg.	neg.	nd	nd	nd
CG32146	*dlp*	1	neg.	neg.	nd	nd	nd
CG32176	CG32176	2	neg.	neg.	nd	nd	nd
CG32177	CG32177	1	neg.	neg.	0	0	0
CG32180	*Eip74EF*	1	neg.	neg.	nd	nd	nd
CG32211	*Taf6*	1	pos.	pos.	34.2	3	10.8
CG32219	CG32219	1	neg.	neg.	nd	nd	nd
CG32225	CG32225	2	neg.	neg.	nd	nd	nd
CG3223	CG3223	2	neg.	neg.	nd	nd	nd
CG3227	CG3227	1	pos.	pos.	4.5	2	0

CG3234	*tim*	2	neg.	neg.	nd	nd	nd
CG32356	*ImpE1*	2	neg.	pos.	0	4.5	1.6
CG32417	*Myt1*	1	neg.	neg.	nd	nd	nd
CG32443	*Pc*	2	neg.	neg.	nd	nd	nd
CG32523	CG32523	2	neg.	pos.	0	0	0
CG3265	*Eb1*	1	neg.	pos.	0	0	0
CG32654	*Sec16*	2	neg.	neg.	nd	nd	nd
CG32656	*Muc11A*	2	neg.	neg.	nd	nd	nd
CG32659	*Ten-a*	1	neg.	neg.	nd	nd	nd
CG32659	*Ten-a*	1	neg.	neg.	nd	nd	nd
CG32676	CG32676	1	neg.	neg.	nd	nd	nd
CG32683	CG32683	2	neg.	neg.	nd	nd	nd
CG32685	CG32685	2	neg.	neg.	nd	nd	nd
CG32694	CG32694	2	pos.	neg.	nd	nd	nd
CG32697	*l(1)G0232*	2	neg.	neg.	nd	nd	nd
CG32698	CG32698	1	pos.	neg.	0	0	0
CG32700	CG32700	2	pos.	pos.	0	0	0
CG32703	CG32703	1	neg.	neg.	nd	nd	nd
CG32703	CG32703	2	neg.	pos.	0	0	0
CG32717	*sdt*	1	neg.	neg.	nd	nd	nd
CG32721	*NELF-B*	2	neg.	pos.	0	1.1	0
CG3274	*Bap170*	2	pos.	neg.	0	0	2
CG32743	*Smg1*	2	neg.	neg.	nd	nd	nd
CG32767	CG32767	1	pos.	pos.	40	1.8	leth
CG32843	*Dh31-R1*	2	neg.	neg.	nd	nd	nd
CG32858	*sn*	1	neg.	neg.	nd	nd	nd
CG3287	CG3287	1	neg.	neg.	nd	nd	nd
CG3289	*Ptpa*	1	neg.	neg.	nd	nd	nd
CG32918	CG15892	1	neg.	neg.	nd	nd	nd
CG32918	CG15891	1	neg.	pos.	0	0	nd
CG3298	*JhI-1*	1	neg.	neg.	nd	nd	nd
CG3307	*pr-set7*	1	neg.	neg.	nd	nd	nd
CG33070	*Sxl*	1	pos.	neg.	0	2	1.2
CG3309	CG3309	1	neg.	neg.	nd	nd	nd
CG3314	*RpL7A*	2	pos.	neg.	0	0	0
CG33156	CG33156	2	pos.	pos.	0	0	4
CG3319	*Cdk7*	1	neg.	neg.	nd	nd	nd
CG33196	*dp*	1	neg.	neg.	nd	nd	nd
CG33203	CG33203	1	neg.	neg.	nd	nd	nd

CG33261	*Trl*	1	pos.	neg.	0	0	0
CG3327	*E23*	1	pos.	neg.	13.1	11.4	27.6
CG3329	*Prosbeta2*	1	neg.	neg.	nd	nd	nd
CG33297	*CSN8*	1	neg.	neg.	nd	nd	nd
CG33466	*Fs*	1	neg.	neg.	nd	nd	nd
CG33517	*D2R*	1	neg.	neg.	nd	nd	nd
CG3354	*Mst77F*	2	neg.	pos.	0	4.7	26.9
CG33714	CG33714	1	neg.	neg.	nd	nd	nd
CG3373	*Hmu*	2	neg.	neg.	nd	nd	nd
CG33934	CG33934	1	neg.	neg.	nd	nd	nd
CG33950	*trol*	2	neg.	neg.	nd	nd	nd
CG33962	*Cp7Fa*	2	pos.	pos.	10.3	0	leth
CG33969	CG33969	1	neg.	neg.	nd	nd	nd
CG34104	CG34104	2	neg.	neg.	nd	nd	nd
CG3412	*slmb*	2	neg.	neg.	nd	nd	nd
CG3428	*pall*	2	neg.	neg.	nd	nd	nd
CG3430	CG3430	1	neg.	pos.	0	0	0
CG34347	CG34347	2	pos.	neg.	0	3.1	0
CG34354	CG34354	2	neg.	neg.	nd	nd	nd
CG34354	CG34354	1	pos.	neg.	0	0	0.9
CG34357	CG34357	2	pos.	neg.	1.6	0	0
CG34384	CG34384	2	neg.	neg.	nd	nd	nd
CG34411	CG34411	1	neg.	neg.	nd	nd	nd
CG3466	*Cyp4d2*	2	neg.	neg.	nd	nd	nd
CG3476	CG3476	1	neg.	pos.	0	0	0
CG3509	CG3509	1	neg.	neg.	nd	nd	nd
CG3522	*Start1*	1	neg.	pos.	2.4	0	0
CG3599	*Btd*	2	pos.	pos.	2	4	1.6
CG3604	CG3604	2	neg.	pos.	2	0	2
CG3613	*qkr58E-1*	1	neg.	neg.	nd	nd	nd
CG3618	CG3618	1	neg.	neg.	nd	nd	nd
CG3632	CG3632	2	neg.	neg.	nd	nd	nd
CG3634	CG3634	1	neg.	neg.	nd	nd	nd
CG3688	*l(2)35Bd*	2	neg.	neg.	nd	nd	nd
CG3700	CG3700	1	pos.	neg.	0	0	1.6
CG3704	CG3704	1	neg.	neg.	nd	nd	nd
CG3705	*aay*	1	neg.	neg.	nd	nd	nd
CG3723	*Dhc93AB*	1	neg.	neg.	nd	nd	nd
CG3729	CG3729	2	pos.	neg.	2.8	0	0

CG3739	CG3739	2	neg.	neg.	nd	nd	nd
CG3759	CG3759	2	neg.	neg.	nd	nd	nd
CG3763	*Fbp2*	2	pos.	neg.	0	4	1.2
CG3798	*Nmda1*	1	neg.	neg.	nd	nd	nd
CG3801	*Acp76A*	2	neg.	neg.	nd	nd	nd
CG3803	CG3803	2	neg.	neg.	nd	nd	nd
CG3809	CG3809	2	pos.	neg.	0	0	0
CG3811	*Oatp30B*	1	neg.	neg.	nd	nd	nd
CG3842	CG3842	1	neg.	neg.	nd	nd	nd
CG3884	CG3884	2	neg.	neg.	nd	nd	nd
CG3886	*Psc*	2	pos.	pos.	4.7	0	0
CG3887	CG3887	2	neg.	pos.	0	0	0
CG3889	*CSN1b*	1	pos.	pos.	10.3	3.6	3.8
CG3897	*blot*	1	neg.	neg.	nd	nd	nd
CG3898	CG3898	1	neg.	pos.	0	0	4.9
CG3902	CG3902	1	neg.	pos.	0	3.8	0
CG3906	CG3906	2	neg.	neg.	nd	nd	nd
CG3915	*Drl-2*	1	neg.	neg.	nd	nd	nd
CG3921	CG3921	1	neg.	neg.	nd	nd	nd
CG3937	*cher*	1	neg.	neg.	nd	nd	nd
CG3954	*csw*	1	neg.	neg.	nd	nd	nd
CG3969	*PR2*	1	pos.	neg.	0	0	3.1
CG3975	CG3975	2	neg.	neg.	nd	nd	nd
CG4001	*Pfk*	1	neg.	neg.	nd	nd	nd
CG4006	*Akt1*	2	pos.	neg.	nd	nd	nd
CG4007	*Nrk*	1	neg.	neg.	nd	nd	nd
CG4012	*gek*	2	neg.	neg.	nd	nd	nd
CG4032	*Abl*	1	neg.	neg.	0	0	0
CG40398	CG17787	2	neg.	neg.	nd	nd	nd
CG4041	CG4041	1	neg.	neg.	nd	nd	nd
CG40410	*Alg-2*	1	neg.	neg.	nd	nd	nd
CG40411	*Parp*	2	neg.	neg.	nd	nd	nd
CG4070	*Tis11*	1	neg.	neg.	nd	nd	nd
CG4073	CG4073	1	neg.	neg.	nd	nd	nd
CG4080	CG4080	1	neg.	neg.	nd	nd	nd
CG4095	CG4095	2	neg.	neg.	nd	nd	nd
CG4097	*Pros26*	1	neg.	neg.	nd	nd	nd
CG4111	*RpL35*	2	neg.	neg.	nd	nd	nd
CG4140	CG4140	1	neg.	neg.	nd	nd	nd

CG4151	CG4151	2	neg.	neg.	nd	nd	nd
CG4159	CG4159	1	neg.	neg.	nd	nd	nd
CG4161	CG4161	2	neg.	pos.	0	0	0
CG4163	*Cyp303a1*	2	neg.	neg.	nd	nd	nd
CG4164	CG4164	1	neg.	neg.	nd	nd	nd
CG4167	*Hsp67Ba*	1	neg.	pos.	2.5	0	nd
CG4181	*GstD2*	2	neg.	neg.	nd	nd	nd
CG4187	CG34411	1	neg.	neg.	nd	nd	nd
CG4192	*kek3*	1	neg.	neg.	nd	nd	nd
CG4200	*sl*	1	pos.	neg.	4.2	0	0
CG42233	CG42233	2	neg.	neg.	nd	nd	nd
CG42244	*Octbeta3R*	2	neg.	pos.	0	0	0
CG42271	CG42271	2	neg.	neg.	nd	nd	nd
CG42273	*mnb*	2	neg.	neg.	nd	nd	nd
CG42303	CG42303	2	neg.	neg.	nd	nd	nd
CG42335	CG42335	1	neg.	neg.	nd	nd	nd
CG42337	CG42337	2	neg.	neg.	nd	nd	nd
CG42348	CG42348	1	neg.	neg.	nd	nd	nd
CG42348	CG42348	1	neg.	neg.	nd	nd	nd
CG42400	CG42400	1	neg.	neg.	nd	nd	nd
CG42534	CG42534	2	neg.	neg.	nd	nd	nd
CG42541	CG42541	2	neg.	neg.	nd	nd	nd
CG4257	*Stat92E*	1	neg.	neg.	nd	nd	nd
CG42573	CG42573	1	neg.	neg.	nd	nd	nd
CG42574	CG42574	2	neg.	neg.	nd	nd	nd
CG42613	CG42613	1	neg.	neg.	nd	nd	nd
CG4262	*elav*	1	pos.	pos.	0	0	0
CG4267	CG4267	2	neg.	neg.	nd	nd	nd
CG4289	CG4289	2	neg.	neg.	nd	nd	nd
CG4299	*Set*	1	neg.	neg.	nd	nd	nd
CG4303	*Bap60*	2	neg.	neg.	nd	nd	nd
CG4314	*st*	2	neg.	neg.	nd	nd	nd
CG4323	CG4323	2	neg.	neg.	nd	nd	nd
CG4336	*rux*	2	neg.	neg.	nd	nd	nd
CG4349	*Fer3HCH*	1	neg.	neg.	nd	nd	nd
CG4353	*hep*	2	pos.	pos.	0	2.8	0
CG4356	*mAcR-60C*	2	neg.	neg.	nd	nd	nd
CG4360	CG4360	1	neg.	neg.	nd	nd	nd
CG4390	CG4390	2	neg.	neg.	nd	nd	nd

CG4396	*fne*	1	pos.	neg.	25	1.5	10.8
CG4404	CG4404	1	neg.	neg.	nd	nd	nd
CG4407	CG4407	2	pos.	neg.	30	2	19
CG4421	*GstD8*	1	neg.	neg.	nd	nd	nd
CG4423	*GstD6*	1	pos.	neg.	0	2.2	0
CG4424	CG4424	2	neg.	pos.	0	0	0
CG4459	CG4459	1	neg.	neg.	nd	nd	nd
CG4465	CG4465	1	neg.	neg.	nd	nd	nd
CG4472	*Idgf1*	2	pos.	neg.	0	0	0
CG4482	*mol*	1	neg.	neg.	nd	nd	nd
CG4486	*Cyp9b2*	2	neg.	neg.	nd	nd	nd
CG4491	*noc*	2	neg.	neg.	nd	nd	nd
CG4521	*mthl1*	1	neg.	neg.	nd	nd	nd
CG4527	*slik*	2	neg.	neg.	nd	nd	nd
CG4550	*ninaE*	2	neg.	neg.	nd	nd	nd
CG4551	*smi35A*	1	neg.	neg.	nd	nd	nd
CG4552	CG4552	1	neg.	neg.	nd	nd	nd
CG4562	CG4562	1	neg.	neg.	nd	nd	nd
CG4574	*Plc21C*	1	neg.	neg.	nd	nd	nd
CG4579	*Nup154*	1	neg.	neg.	nd	nd	nd
CG4582	CG4582	1	neg.	neg.	nd	nd	nd
CG4587	CG4587	2	neg.	neg.	nd	nd	nd
CG4607	CG4607	1	neg.	neg.	nd	nd	nd
CG4631	CG4631	1	pos.	neg.	0	0	0
CG4637	*hh*	1	neg.	neg.	nd	nd	nd
CG4647	*mRpL49*	1	neg.	neg.	nd	nd	nd
CG4659	*Srp54k*	2	pos.	pos.	31.7	0	leth
CG4661	CG4661	2	pos.	neg.	nd	nd	nd
CG4686	CG4686	1	neg.	neg.	nd	nd	nd
CG4694	*her*	2	pos.	neg.	0	0	0
CG4697	*CSN1a*	2	neg.	pos.	0	0	0
CG4703	*Arc42*	1	neg.	neg.	nd	nd	nd
CG4707	CG4707	2	pos.	pos.	0	1.2	9.7
CG4712	CG4712	2	neg.	neg.	nd	nd	nd
CG4715	*Iris*	2	neg.	neg.	nd	nd	nd
CG4727	*bol*	2	neg.	neg.	nd	nd	nd
CG4755	*RhoGAP92B*	1	neg.	neg.	nd	nd	nd
CG4757	CG4757	1	neg.	neg.	nd	nd	nd
CG4784	*Cpr72Ec*	2	neg.	neg.	nd	nd	nd

CG4793	CG4793	2	pos.	pos.	0	2	1.2
CG4812	*Ser8*	2	pos.	neg.	0	0	4.1
CG4817	*Ssrp*	1	pos.	pos.	1.5	5.2	1
CG4822	CG4822	1	neg.	neg.	nd	nd	nd
CG4830	CG4830	2	neg.	neg.	nd	nd	nd
CG4838	*beat-Ic*	1	neg.	neg.	nd	nd	nd
CG4839	CG4839	2	neg.	neg.	nd	nd	nd
CG4852	*Sras*	1	neg.	pos.	0	0	0
CG4854	CG4854	1	neg.	neg.	nd	nd	nd
CG4858	CG4858	1	neg.	neg.	nd	nd	nd
CG4863	*RpL3*	2	neg.	neg.	nd	nd	nd
CG4904	*Pros35*	1	pos.	pos.	2.8	5	leth
CG4914	CG4914	1	neg.	neg.	nd	nd	nd
CG4919	*Gclm*	2	neg.	pos.	0	0	0
CG4926	*Ror*	2	neg.	neg.	nd	nd	nd
CG4955	CG4955	1	neg.	neg.	nd	nd	nd
CG4965	*twe*	1	neg.	neg.	nd	nd	nd
CG4995	CG4995	2	neg.	pos.	0	1.2	0
CG5044	CG5044	1	pos.	neg.	0	3.3	3.7
CG5059	CG5059	1	neg.	neg.	nd	nd	nd
CG5069	*croc*	1	pos.	neg.	0	4	0
CG5091	CG5091	1	neg.	neg.	nd	nd	nd
CG5092	*Tor*	1	neg.	neg.	nd	nd	nd
CG5094	*Sgt*	1	neg.	neg.	nd	nd	nd
CG5118	CG5118	2	neg.	neg.	nd	nd	nd
CG5147	CG5147	1	neg.	neg.	nd	nd	nd
CG5171	CG5171	1	neg.	neg.	nd	nd	nd
CG5178	*Act88F*	2	neg.	neg.	nd	nd	nd
CG5180	CG5180	2	neg.	neg.	nd	nd	nd
CG5181	CG5181	1	neg.	pos.	0	0	0
CG5203	*CHIP*	2	neg.	neg.	nd	nd	nd
CG5220	CG5220	1	neg.	pos.	0	0	0
CG5222	CG5222	1	pos.	neg.	3.9	0	18.5
CG5232	*Sas*	1	neg.	neg.	nd	nd	nd
CG5315	CG5315	2	neg.	neg.	nd	nd	nd
CG5325	CG5325	1	neg.	neg.	nd	nd	nd
CG5358	*Art4*	1	neg.	neg.	nd	nd	nd
CG5371	*RnrL*	1	neg.	pos.	0	0.9	19.8
CG5378	*Rpn7*	2	neg.	neg.	nd	nd	nd

CG5390	CG5390	2	neg.	neg.	nd	nd	nd
CG5427	*Oatp33Ea*	2	pos.	neg.	4	0	0
CG5427	*Oatp33Ea*	1	neg.	neg.	nd	nd	nd
CG5439	CG5439	2	pos.	neg.	0	0	nd
CG5475	*Mpk2*	1	neg.	neg.	nd	nd	nd
CG5483	*Lrrk*	1	neg.	neg.	nd	nd	nd
CG5494	*Cpr92F*	1	pos.	pos.	3.4	4.3	nd
CG5519	*Prp19*	2	pos.	neg.	9.3	0	0
CG5528	*Toll-9*	1	neg.	neg.	nd	nd	nd
CG5543	CG5543	1	neg.	neg.	nd	nd	nd
CG5550	CG5550	1	pos.	pos.	0	0	0
CG5561	CG5561	2	neg.	neg.	nd	nd	nd
CG5585	CG5585	2	neg.	neg.	nd	nd	nd
CG5596	*Mlc1*	1	neg.	neg.	nd	nd	nd
CG5597	CG5597	2	neg.	neg.	nd	nd	nd
CG5604	CG5604	1	neg.	pos.	0	4.8	0
CG5605	*eRF1*	2	pos.	neg.	15.9	0	16.7
CG5611	CG5611	2	neg.	neg.	nd	nd	nd
CG5612	CG5612	1	neg.	neg.	nd	nd	nd
CG5637	*nos*	2	neg.	neg.	nd	nd	nd
CG5640	*Utx*	2	neg.	neg.	0	0	0
CG5641	CG5641	3	neg.	neg.	nd	nd	nd
CG5643	*wdb*	1	neg.	neg.	nd	nd	nd
CG5649	*kin17*	1	pos.	pos.	30	1.3	1.3
CG5661	*Sema-5c*	2	neg.	neg.	nd	nd	nd
CG5671	*Pten*	2	neg.	neg.	nd	nd	nd
CG5680	*bsk*	1	neg.	neg.	nd	nd	nd
CG5703	CG5703	1	neg.	neg.	nd	nd	nd
CG5704	CG5704	1	neg.	neg.	nd	nd	nd
CG5707	CG5707	1	neg.	pos.	0	0	0
CG5720	CG5720	2	neg.	neg.	nd	nd	nd
CG5737	*dmrt93B*	1	pos.	neg.	4.8	0	3.3
CG5758	CG5758	2	pos.	neg.	0	0	0
CG5783	CG5783	2	neg.	neg.	nd	nd	nd
CG5784	*Mapmodulin*	1	neg.	neg.	nd	nd	nd
CG5786	*ppan*	1	pos.	neg.	31.3	16	45.7
CG5789	CG5789	1	neg.	neg.	nd	nd	nd
CG5790	CG5790	1	pos.	pos.	0	0	0
CG5807	CG5807	2	neg.	neg.	nd	nd	nd

CG5818	*mRpL4*	2	neg.	neg.	nd	nd	nd
CG5838	*Dref*	2	pos.	neg.	13.6	2.5	100
CG5847	CG5847	1	neg.	neg.	nd	nd	nd
CG5848	*cact*	1	pos.	pos.	12	4.4	25.4
CG5853	CG5853	2	neg.	pos.	0	0	4
CG5854	CG5854	1	neg.	neg.	nd	nd	nd
CG5855	*cni*	1	neg.	neg.	nd	nd	nd
CG5872	CG5872	1	neg.	neg.	nd	nd	nd
CG5877	CG5877	1	neg.	neg.	nd	nd	nd
CG5905	*Nep1*	2	neg.	neg.	nd	nd	nd
CG5910	CG5910	1	pos.	neg.	0	3.4	1.3
CG5925	*desat2*	2	neg.	pos.	0	0	3.1
CG5938	CG5938	2	neg.	neg.	nd	nd	nd
CG5939	*Prm*	2	neg.	neg.	nd	nd	nd
CG5953	CG5953	1	neg.	neg.	nd	nd	nd
CG5968	CG5968	2	neg.	neg.	nd	nd	nd
CG5974	*pll*	1	neg.	neg.	nd	nd	nd
CG5991	CG5991	2	neg.	neg.	nd	nd	nd
CG5993	*os*	2	neg.	neg.	nd	nd	nd
CG5999	CG5999	2	neg.	neg.	nd	nd	nd
CG6012	CG6012	1	neg.	neg.	nd	nd	nd
CG6027	*cdi*	1	neg.	neg.	nd	nd	nd
CG6036	CG6036	1	neg.	neg.	nd	nd	nd
CG6048	CG6048	1	neg.	neg.	nd	nd	nd
CG6054	*Su(fu)*	1	neg.	neg.	nd	nd	nd
CG6121	*Tip60*	1	pos.	neg.	14.3	0	1.8
CG6122	*piwi*	2	neg.	neg.	nd	nd	nd
CG6143	*Pep*	2	neg.	neg.	nd	nd	nd
CG6151	CG6151	1	neg.	neg.	nd	nd	nd
CG6182	CG6182	2	neg.	neg.	nd	nd	nd
CG6188	CG6188	2	neg.	neg.	nd	nd	nd
CG6189	*l(1)1Bi*	1	neg.	neg.	nd	nd	nd
CG6195	CG6195	1	neg.	neg.	nd	nd	nd
CG6197	CG6197	2	pos.	pos.	45	0	leth
CG6199	CG6199	1	neg.	neg.	nd	nd	nd
CG6207	*GlcAT-P*	2	neg.	neg.	nd	nd	nd
CG6214	*MRP*	1	neg.	neg.	nd	nd	nd
CG6220	CG6220	2	neg.	neg.	nd	nd	nd
CG6225	CG6225	2	neg.	neg.	nd	nd	nd

CG6226	*FK506-bp1*	1	pos.	pos.	2	3.8	1.2
CG6238	*ssh*	2	neg.	neg.	nd	nd	nd
CG6251	*Nup62*	2	neg.	neg.	nd	nd	nd
CG6255	CG6255	1	neg.	neg.	nd	nd	nd
CG6273	*Eip74EF*	1	neg.	neg.	nd	nd	nd
CG6280	CG6280	1	neg.	neg.	nd	nd	nd
CG6282	CG6282	2	neg.	neg.	nd	nd	nd
CG6284	*Sirt6*	1	neg.	neg.	nd	nd	nd
CG6289	CG6289	2	neg.	neg.	nd	nd	nd
CG6292	*CycT*	2	pos.	neg.	33.3	0	10
CG6298	*Jon74E*	1	neg.	neg.	nd	nd	nd
CG6311	*Edc3*	2	pos.	neg.	20.5	4	100
CG6322	CG6322	1	pos.	neg.	4.5	5	37.1
CG6333	CG6333	1	neg.	neg.	nd	nd	nd
CG6337	CG6337	3	neg.	neg.	nd	nd	nd
CG6355	*fab1*	2	neg.	neg.	nd	nd	nd
CG6380	CG6380	2	neg.	neg.	nd	nd	nd
CG6386	*ball*	2	neg.	neg.	nd	nd	nd
CG6386	*ball*	1	neg.	neg.	nd	nd	nd
CG6392	*cmet*	2	neg.	neg.	nd	nd	nd
CG6426	CG6426	2	neg.	neg.	nd	nd	nd
CG6429	CG6429	2	neg.	neg.	nd	nd	nd
CG6434	CG6434	1	pos.	neg.	0	0	0
CG6451	*blue*	1	neg.	neg.	nd	nd	nd
CG6459	CG6459	1	pos.	neg.	0	0	0
CG6470	CG6470	1	neg.	pos.	0	0	0
CG6480	CG6480	2	neg.	neg.	nd	nd	nd
CG6483	*Jon65Aiii*	1	neg.	neg.	nd	nd	nd
CG6489	*Hsp70Bc*	1	pos.	pos.	26.4	15.5	68.1
CG6495	CG6495	1	neg.	neg.	nd	nd	nd
CG6495	CG6495	2	neg.	neg.	nd	nd	nd
CG6498	CG6498	2	neg.	neg.	nd	nd	nd
CG6501	*ns2*	2	neg.	neg.	nd	nd	nd
CG6502	*E(z)*	2	neg.	neg.	nd	nd	nd
CG6518	*inaC*	3	neg.	neg.	nd	nd	nd
CG6523	CG6523	2	neg.	neg.	nd	nd	nd
CG6524	*Cp19*	2	neg.	neg.	nd	nd	nd
CG6525	*pps*	2	neg.	neg.	nd	nd	nd
CG6540	CG6540	1	neg.	neg.	nd	nd	nd

CG6551	*fu*	1	neg.	neg.	nd	nd	nd
CG6571	*rdgC*	2	neg.	neg.	nd	nd	nd
CG6582	*Aac11*	1	neg.	neg.	nd	nd	nd
CG6597	CG6597	1	pos.	neg.	0	0	0
CG6617	CG6617	1	neg.	pos.	0	0	0
CG6637	*lsn*	1	neg.	neg.	nd	nd	nd
CG6639	CG6639	2	neg.	neg.	nd	nd	nd
CG6647	*porin*	1	neg.	neg.	nd	nd	nd
CG6672	CG6672	2	neg.	neg.	nd	nd	nd
CG6673	CG6673	1	neg.	neg.	nd	nd	nd
CG6704	CG6704	1	neg.	neg.	nd	nd	nd
CG6715	*KP78a*	2	neg.	neg.	nd	nd	nd
CG6739	CG6739	1	neg.	neg.	nd	nd	nd
CG6745	CG6745	2	neg.	pos.	0	0	2.9
CG6750	CG6750	1	neg.	pos.	4	0	0
CG6789	CG6789	1	neg.	neg.	0	0	0
CG6794	*Dif*	2	neg.	neg.	nd	nd	nd
CG6818	*MESR6*	1	neg.	neg.	nd	nd	nd
CG6819	*mbo*	2	neg.	neg.	nd	nd	nd
CG6831	*rhea*	1	neg.	neg.	nd	nd	nd
CG6846	*RpL26*	2	pos.	neg.	14.3	0	leth
CG6854	CG6854	1	neg.	neg.	nd	nd	nd
CG6870	CG6870	1	neg.	pos.	0	0	0
CG6885	CG6885	1	pos.	pos.	0	2	0
CG6919	*oa2*	2	neg.	neg.	nd	nd	nd
CG6950	CG6950	2	neg.	neg.	0	0	0
CG6951	CG6951	2	neg.	neg.	nd	nd	nd
CG6962	CG6962	2	pos.	neg.	0	0	1.8
CG6963	*gish*	1	neg.	neg.	nd	nd	nd
CG6964	*Gug*	1	neg.	neg.	nd	nd	nd
CG6977	*Cad87A*	1	pos.	pos.	0	0	0
CG6998	*ctp*	1	neg.	neg.	nd	nd	nd
CG6999	CG6999	1	neg.	neg.	nd	nd	nd
CG7004	*fwd*	1	neg.	neg.	nd	nd	nd
CG7014	*RpS5b*	1	pos.	pos.	8.2	3.6	0
CG7017	CG7017	2	neg.	neg.	nd	nd	nd
CG7033	CG7033	2	pos.	pos.	39	1.4	2
CG7049	CG7049	1	neg.	neg.	nd	nd	nd
CG7091	CG7091	2	pos.	neg.	0	0	0.9

CG7095	CG42450	1	neg.	neg.	nd	nd	nd
CG7097	*hppy*	1	neg.	neg.	nd	nd	nd
CG7099	CG7099	2	neg.	neg.	nd	nd	nd
CG7100	*CadN*	1	neg.	neg.	nd	nd	nd
CG7133	CG7133	1	neg.	neg.	nd	nd	nd
CG7134	*cdc14*	2	neg.	neg.	nd	nd	nd
CG7148	CG7148	1	neg.	neg.	nd	nd	nd
CG7157	*Acp36DE*	1	neg.	pos.	0	0	0
CG7168	CG7168	2	neg.	pos.	0	2.2	0
CG7170	*Jon66Cii*	2	neg.	pos.	0	0	nd
CG7171	*Uro*	2	pos.	neg.	0	0	0
CG7177	CG7177	1	neg.	neg.	nd	nd	nd
CG7200	CG7200	2	neg.	neg.	0	0	0
CG7219	*Spn28D*	2	neg.	neg.	nd	nd	nd
CG7241	*Cyp304a1*	2	neg.	neg.	nd	nd	nd
CG7252	CG7252	1	neg.	neg.	nd	nd	nd
CG7257	*Rpt4R*	2	neg.	pos.	4.9	1	0
CG7275	CG7275	2	pos.	neg.	0	0	0
CG7290	CG7290	1	pos.	neg.	0	0	0
CG7293	*Klp68D*	1	neg.	neg.	nd	nd	nd
CG7300	CG7300	1	pos.	neg.	0	0	0
CG7300	CG7300	1	pos.	neg.	1.1	0	0
CG7306	*obst-F*	1	neg.	neg.	nd	nd	nd
CG7325	*Eig71Ek*	1	neg.	neg.	nd	nd	nd
CG7328	CG7328	2	neg.	neg.	nd	nd	nd
CG7337	CG7337	1	neg.	neg.	nd	nd	nd
CG7351	*PCID2*	2	neg.	neg.	nd	nd	nd
CG7352	CG7352	2	pos.	neg.	0	0	1.3
CG7357	CG7357	1	neg.	neg.	nd	nd	nd
CG7358	CG7358	1	neg.	neg.	nd	nd	nd
CG7362	CG7362	2	neg.	neg.	nd	nd	nd
CG7365	CG7365	1	neg.	neg.	nd	nd	nd
CG7365	CG7365	1	neg.	neg.	nd	nd	nd
CG7375	CG7375	1	neg.	neg.	nd	nd	nd
CG7393	*p38b*	1	neg.	neg.	0	0	4
CG7407	CG7407	2	pos.	neg.	1.4	0	2.1
CG7411	*ort*	1	neg.	neg.	nd	nd	nd
CG7415	*DppIII*	1	neg.	neg.	nd	nd	nd
CG7442	CG7442	1	neg.	neg.	nd	nd	nd

CG7461	CG7461	1	neg.	neg.	nd	nd	nd
CG7486	*Dredd*	2	neg.	neg.	nd	nd	nd
CG7507	*Dhc64C*	1	neg.	pos.	nd	nd	nd
CG7524	*Src64B*	2	neg.	neg.	nd	nd	nd
CG7542	CG7542	1	neg.	neg.	nd	nd	nd
CG7555	*Nedd4*	2	neg.	neg.	0	0	0
CG7569	*DopR2*	2	neg.	neg.	nd	nd	nd
CG7582	CG7582	2	neg.	neg.	nd	nd	nd
CG7602	*DNApol-iota*	2	pos.	neg.	0	0	0
CG7619	*Pros54*	2	neg.	neg.	nd	nd	nd
CG7632	CG7632	1	neg.	neg.	nd	nd	nd
CG7644	*beat-Ib*	1	neg.	neg.	nd	nd	nd
CG7646	CG7646	1	neg.	pos.	0	0	nd
CG7693	*fray*	1	neg.	neg.	nd	nd	nd
CG7717	*Mekk1*	2	neg.	neg.	nd	nd	nd
CG7725	*rogdi*	1	pos.	neg.	0	0	0
CG7745	CG7745	2	pos.	pos.	0	0	0
CG7756	*Hsc70-2*	2	neg.	pos.	0	0	2.9
CG7757	CG7757	1	pos.	neg.	50.8	6	leth
CG7758	*ppl*	2	pos.	pos.	0	0	2
CG7765	*Khc*	1	neg.	neg.	nd	nd	nd
CG7770	CG7770	2	neg.	neg.	nd	nd	nd
CG7777	CG7777	2	pos.	neg.	0	0	2
CG7826	*Gmap*	1	neg.	neg.	nd	nd	nd
CG7831	*ncd*	1	neg.	neg.	nd	nd	nd
CG7838	*BubR1*	2	neg.	neg.	nd	nd	nd
CG7843	CG7843	2	neg.	pos.	0	0	0
CG7845	CG7845	1	pos.	neg.	3.6	0	48.6
CG7873	*Src42A*	2	pos.	neg.	0	0	3.6
CG7885	*RpII33*	1	neg.	pos.	0	10	95
CG7904	*put*	2	pos.	neg.	0	0	0
CG7918	CG7918	1	pos.	pos.	0	0	nd
CG7927	CG7927	1	neg.	neg.	nd	nd	nd
CG7940	*Arp5*	2	neg.	neg.	nd	nd	nd
CG7959	*Bgb*	2	pos.	neg.	4.4	0	0
CG7978	*Ac76E*	1	neg.	neg.	nd	nd	nd
CG7996	*snk*	1	neg.	neg.	nd	nd	nd
CG8023	*eIF4E-3*	1	neg.	neg.	nd	nd	nd
CG8025	*Mtr3*	1	pos.	neg.	12.5	0	21.7

CG8049	*Btk29A*	1	neg.	neg.	nd	nd	nd
CG8080	CG8080	1	neg.	neg.	nd	nd	nd
CG8086	CG8086	1	neg.	neg.	nd	nd	nd
CG8090	CG8090	2	neg.	neg.	nd	nd	nd
CG8093	CG8093	2	neg.	neg.	nd	nd	nd
CG8107	*CalpB*	2	neg.	neg.	nd	nd	nd
CG8112	CG8112	2	neg.	neg.	nd	nd	nd
CG8127	*Eip75B*	1	neg.	neg.	nd	nd	nd
CG8134	CG8134	2	pos.	neg.	1.2	0	0
CG8142	CG8142	2	pos.	neg.	2.4	0	0
CG8152	CG8152	1	neg.	neg.	nd	nd	nd
CG8171	*dup*	1	neg.	neg.	nd	nd	nd
CG8173	CG8173	1	pos.	neg.	0	0	0
CG8193	CG8193	1	neg.	neg.	nd	nd	nd
CG8199	CG8199	1	neg.	neg.	nd	nd	nd
CG8203	*Cdk5*	1	neg.	neg.	nd	nd	nd
CG8206	CG8206	2	neg.	neg.	nd	nd	nd
CG8210	*Vha14*	2	neg.	neg.	nd	nd	nd
CG8222	*Pvr*	1	neg.	neg.	nd	nd	nd
CG8224	*babo*	2	neg.	neg.	nd	nd	nd
CG8257	CG8257	1	neg.	pos.	3.2	0	4.8
CG8271	*Sln*	2	neg.	pos.	0	0	1.9
CG8274	*Mtor*	2	neg.	neg.	nd	nd	nd
CG8309	*Tango7*	2	neg.	neg.	nd	nd	nd
CG8319	CG8319	1	neg.	neg.	nd	nd	nd
CG8329	CG8329	1	pos.	pos.	3.3	3.5	1.2
CG8336	CG8336	2	neg.	neg.	nd	nd	nd
CG8355	*sli*	1	neg.	neg.	nd	nd	nd
CG8386	CG8386	2	pos.	neg.	0	0	0
CG8402	*PpD3*	2	neg.	neg.	nd	nd	nd
CG8428	*spin*	2	neg.	neg.	nd	nd	nd
CG8428	*spin*	1	pos.	neg.	0	0	0
CG8444	CG8444	1	neg.	pos.	3	0	10
CG8461	CG8461	2	neg.	neg.	nd	nd	nd
CG8470	*mRpS30*	1	pos.	pos.	0	7.1	94.3
CG8493	*Den1*	1	neg.	neg.	nd	nd	nd
CG8507	CG8507	2	neg.	neg.	nd	nd	nd
CG8511	*Cpr49Ag*	1	neg.	neg.	nd	nd	nd
CG8515	*Cpr49Ah*	1	pos.	neg.	0	0	0

CG8525	CG8525	1	pos.	neg.	0	0	0
CG8550	CG8550	2	neg.	neg.	nd	nd	nd
CG8561	*conv*	1	neg.	neg.	nd	nd	nd
CG8565	CG8565	1	neg.	neg.	nd	nd	nd
CG8571	*smid*	1	neg.	neg.	nd	nd	nd
CG8580	*akirin*	1	neg.	neg.	nd	nd	nd
CG8586	CG8586	2	neg.	pos.	0	0	0
CG8590	*Klp3A*	1	neg.	neg.	nd	nd	nd
CG8595	*Toll-7*	2	pos.	pos.	8.3	5	50
CG8599	*Su(var)3-7*	1	neg.	neg.	nd	nd	nd
CG8657	*Dgkepsilon*	2	neg.	neg.	nd	nd	nd
CG8701	CG8701	1	pos.	neg.	0	0	0
CG8711	*cul-4*	1	pos.	pos.	46	39.4	77.1
CG8719	*Odc2*	1	neg.	neg.	nd	nd	nd
CG8722	*Nup44A*	1	pos.	neg.	0	0	0
CG8725	*CSN4*	2	pos.	neg.	nd	nd	nd
CG8727	*cyc*	1	neg.	neg.	nd	nd	nd
CG8729	*rnh1*	1	neg.	neg.	nd	nd	nd
CG8738	CG8738	2	neg.	neg.	nd	nd	nd
CG8742	*Gyc76C*	2	neg.	neg.	0	0	0
CG8756	*verm*	2	pos.	pos.	nd	nd	nd
CG8767	*mos*	2	neg.	neg.	nd	nd	nd
CG8780	*tey*	2	pos.	neg.	4	0	0
CG8789	*wnd*	2	neg.	neg.	nd	nd	nd
CG8805	*wun2*	1	neg.	neg.	nd	nd	nd
CG8827	*Ance*	1	neg.	neg.	nd	nd	nd
CG8867	*Jon25Bi*	1	neg.	pos.	0	3.9	0
CG8871	*Jon25Biii*	2	neg.	neg.	nd	nd	nd
CG8878	CG8878	2	pos.	neg.	12.2	0	3.9
CG8881	*skpB*	1	neg.	neg.	0	0	0
CG8888	CG8888	1	neg.	neg.	nd	nd	nd
CG8896	*18w*	2	neg.	neg.	nd	nd	nd
CG8905	*Sod2*	1	neg.	neg.	nd	nd	nd
CG8909	CG8909	2	neg.	neg.	nd	nd	nd
CG8913	*Irc*	1	neg.	pos.	0	3.6	nd
CG8915	CG8915	1	neg.	pos.	0	0	0
CG8952	CG8952	2	neg.	neg.	nd	nd	nd
CG8954	*Smg5*	1	neg.	neg.	nd	nd	nd
CG8967	*otk*	2	neg.	neg.	nd	nd	nd

CG8985	*DmsR-1*	1	neg.	neg.	nd	nd	nd
CG8989	*His3.3B*	1	neg.	neg.	nd	nd	nd
CG9107	CG9107	1	pos.	pos.	7.3	2.5	8.8
CG9114	CG9114	1	neg.	neg.	nd	nd	nd
CG9156	*Pp1-13C*	1	neg.	neg.	nd	nd	nd
CG9172	CG9172	1	neg.	neg.	nd	nd	nd
CG9181	*Ptp61F*	1	neg.	neg.	nd	nd	nd
CG9186	CG9186	2	neg.	neg.	nd	nd	nd
CG9196	*spz6*	1	neg.	neg.	nd	nd	nd
CG9204	*Ate1*	2	neg.	neg.	nd	nd	nd
CG9210	*Ac13E*	1	neg.	neg.	nd	nd	nd
CG9262	*Shal*	2	neg.	neg.	nd	nd	nd
CG9279	CG9279	2	neg.	neg.	nd	nd	nd
CG9299	*Cpr76Bd*	1	neg.	neg.	nd	nd	nd
CG9305	CG9305	1	pos.	pos.	13.9	7.7	leth
CG9311	*mop*	1	pos.	neg.	1.7	0	2
CG9327	*Pros29*	1	neg.	neg.	nd	nd	nd
CG9335	CG9335	2	neg.	neg.	nd	nd	nd
CG9356	CG9356	1	neg.	pos.	0	1.3	0
CG9381	*mura*	1	neg.	neg.	nd	nd	nd
CG9386	CG9386	1	neg.	neg.	nd	nd	nd
CG9391	CG9391	1	neg.	neg.	nd	nd	nd
CG9396	CG9396	1	neg.	neg.	nd	nd	nd
CG9415	*Xbp1*	1	neg.	neg.	nd	nd	nd
CG9433	*Xpd*	2	pos.	neg.	0	0	12.3
CG9441	*Pu*	2	pos.	neg.	3.2	0	22
CG9471	CG9471	1	neg.	neg.	nd	nd	nd
CG9484	*hyd*	1	neg.	neg.	nd	nd	nd
CG9509	CG9509	1	neg.	neg.	nd	nd	nd
CG9533	*rut*	2	neg.	neg.	nd	nd	nd
CG9564	*Try29F*	2	neg.	neg.	nd	nd	nd
CG9565	*Nep3*	1	neg.	neg.	nd	nd	nd
CG9568	CG9568	2	neg.	neg.	nd	nd	nd
CG9579	*AnnX*	1	neg.	neg.	nd	nd	nd
CG9588	CG9588	2	neg.	neg.	nd	nd	nd
CG9589	CG9589	2	neg.	neg.	nd	nd	nd
CG9602	CG9602	1	neg.	neg.	nd	nd	nd
CG9611	*f-cup*	1	neg.	neg.	nd	nd	nd
CG9640	CG9640	2	neg.	pos.	0	0	0

CG9641	CG9641	2	neg.	neg.	nd	nd	nd
CG9663	CG9663	2	pos.	pos.	31	0	0.9
CG9664	CG9664	1	neg.	neg.	nd	nd	nd
CG9672	CG9672	2	pos.	neg.	0	0	1.2
CG9673	CG9673	1	neg.	pos.	0	0	0
CG9675	*spheroide*	2	neg.	neg.	nd	nd	nd
CG9676	CG9676	1	pos.	pos.	2.1	0	0
CG9680	*Dbp73D*	1	neg.	neg.	nd	nd	nd
CG9722	CG9722	1	neg.	neg.	nd	nd	nd
CG9737	CG9737	2	neg.	neg.	nd	nd	nd
CG9738	*Mkk4*	2	neg.	neg.	nd	nd	nd
CG9745	*D1*	1	pos.	pos.	0	0	26.5
CG9749	*Abi*	2	neg.	neg.	nd	nd	nd
CG9774	*Lectin-galC1*	2	neg.	neg.	nd	nd	nd
CG9774	*rok*	2	neg.	neg.	nd	nd	nd
CG9784	CG9784	1	pos.	neg.	0	0	0
CG9805	*eIF3-S10*	2	pos.	pos.	4.3	1.7	0
CG9853	CG9853	1	pos.	neg.	8.4	0	23.1
CG9952	*ppa*	1	pos.	neg.	0	0	0
CG9987	CG9987	1	neg.	neg.	nd	nd	nd
